# Tularemia Antimicrobial Treatment and Prophylaxis: CDC Recommendations for Naturally Acquired Infections and Bioterrorism Response — United States, 2025

**DOI:** 10.15585/mmwr.rr7402a1

**Published:** 2025-10-02

**Authors:** Christina A. Nelson, Dana Meaney-Delman, Shannon Fleck-Derderian, Jessica Winberg, Paul S. Mead

**Affiliations:** ^1^Division of Vector-Borne Diseases, National Center for Emerging and Zoonotic Infectious Diseases, CDC, Fort Collins, Colorado; ^2^Infant Outcomes Monitoring, Research and Prevention Branch, Division of Birth Defects and Infant Disorders, CDC, Atlanta, Georgia; ^3^Alaka`ina Foundation, Fort Collins, Colorado

## Abstract

*This report provides CDC recommendations to U.S. health care providers and preparedness personnel regarding treatment and postexposure prophylaxis (PEP) of tularemia,* *an uncommon but potentially serious disease caused by the gram-negative coccobacillus,* Francisella tularensis. *Tularemia occurs naturally in the United States and other Northern Hemisphere regions. Because* F. tularensis *has a low infectious inoculum, it* *is classified as a potential bioterrorism agent that could infect thousands of persons if intentionally released, requiring rapid, informed decision-making by public health agencies, first responders, and clinicians. To mitigate the effects of a bioterrorism attack, the U.S. government stockpiles medical countermeasures, and the 21st Century Cures Act mandates development of evidence-based guidelines for their use. Since 2001 when guidelines for tularemia treatment and PEP were last published, new animal study data and human clinical data have become available. CDC compiled a broad evidence base by conducting a series of systematic reviews of the literature on human tularemia through 2023, analyzing U.S. surveillance data, gathering outbreak reports and case series, and collecting animal data. During a series of scientific forums, evidence was presented from these investigations and additional data sources to subject matter experts, and individual expert input on proposed recommendations was solicited. The guidelines team then assessed the available evidence, considered different perspectives and feedback shared in the expert forums, and used the Grading of Recommendations, Assessment, Development and Evaluation summary of findings and the Evidence to Decision framework to formulate recommendations based on the balance of benefits and harms. Notable changes include use of a treatment and prophylaxis framework; designation of fluoroquinolones (ciprofloxacin or levofloxacin) and doxycycline as first-line treatment options for outbreaks of any size; identification of third-tier treatment options when first-line and alternative antimicrobials are unavailable or contraindicated for certain patients; and recommendations for neonates, breastfeeding infants, lactating mothers,* *patients with* *immunocompromise, and geriatric patients. These guidelines provide a summary of best practices for treatment and prophylaxis of human tularemia for both naturally occurring disease and after a bioterrorism attack. They do not include information on dispensing medical countermeasures, diagnostic testing, triage decisions, or adjunct treatments for patients with tularemia. Health care providers can use these guidelines to manage patients with naturally occurring infection and, with public health officials, prepare their organizations, clinics, hospitals, and communities to respond to a tularemia mass exposure event.*

## Introduction

Tularemia is a rare but potentially serious disease caused by *Francisella tularensis,* a highly infectious, nonmotile, gram-negative coccobacillus. *F. tularensis* is endemic throughout the Northern Hemisphere, with the majority of annual infections reported from the United States, Turkey, and Northern Europe (*1*,*2*). Approximately 200–300 cases of tularemia, also known as rabbit fever or deer fly fever, are reported in the United States annually; cases have been reported from every state except Hawaii (*3*).

In the United States, two subspecies of *F. tularensis* are linked to human disease. The most common subspecies, *F. tularensis* subspecies *tularensis* (Type A), is found only in North America and is further divided into distinct A1 (East) and A2 (West) subpopulations. Type A1 is more virulent and associated with a higher case-fatality rate (CFR) (*4*–*6*). *F. tularensis* subspecies *holarctica* (Type B) is found throughout the Northern Hemisphere and is responsible for the majority of tularemia cases reported globally. Three biovars within *F. tularensis* subspecies *holarctica* have been described: biovar I in North America and Western Europe, biovar II in Eastern Europe and Asia, and biovar *japonica* strains, primarily found in Japan but also present in China and Turkey. The overall CFR for tularemia is approximately 2%–3% for Type A infections and <1% for Type B infections. Although Type B is linked to a lower CFR, it can cause protracted illness, disability, and substantial morbidity (*1*,*4*,*6*,*7*).

Humans can be exposed to *F. tularensis* through various routes, including bites from arthropods (e.g., ticks, deer flies, and mosquitos), percutaneous exposure through butchering or other contact with infected animals, ingestion of contaminated food and water, contact with contaminated soil or hay, and inhalation of aerosolized particles (*7*,*8*). Although the organism has a low infectious inoculum and human-to-human transmission might be expected, human-to-human transmission is exceedingly rare. In 1926, a woman developed ulceroglandular tularemia after pricking her thumb while tending to an infected ulcer on her son’s ear (*9*). Ulceroglandular infection after performing autopsy has been reported twice, once in 1919 involving Dr. Edward Francis, for whom the bacterium is named (*10*), and again in 1936 when a physician cut her thumb during a postmortem examination (*11*). More recently, three persons became ill after receiving solid organ transplants from an infected donor (*12*). No cases of human-to-human transmission resulting from aerosolized particles have been reported.

Disease manifestation is largely determined by the route of exposure. Ulceroglandular and glandular tularemia, the most common presentations, often are caused by an arthropod bite or inoculation through broken skin when handling infected animal tissues. In certain cases, localized infection can progress via hematogenous spread to more severe systemic disease. Ingestion of *F. tularensis* can cause oropharyngeal tularemia; exposure to the eyes results in oculoglandular disease. Inhaling aerosolized *F. tularensis* leads to pneumonic tularemia, typically the most severe manifestation. Typhoidal tularemia, a systemic form of infection that tends to affect older adults and persons with chronic illness, often lacks localized signs and symptoms, although gastrointestinal symptoms (vomiting and diarrhea), hepatosplenomegaly, or an altered mental state might be present along with signs of sepsis (*3*,*4*,*13*). Less common but potentially severe manifestations of tularemia include meningitis, septic arthritis, osteomyelitis, endocarditis, otitis media, and mastoiditis (*14*–*17*).

Tularemia is treatable with antimicrobial agents, including aminoglycosides, fluoroquinolones, and tetracyclines. Specific clinical manifestations (typhoidal or pneumonic disease) (*4*) and delays in initiation of treatment are associated with worse outcomes (*18*). Beta-lactam antibiotics (including penicillins, cephalosporins, and carbapenems), polymyxins, glycopeptides, and sulfonamides are ineffective for treatment of tularemia, despite the appearance of in vitro susceptibility for certain types of these agents (*1*). Although tularemia vaccines have been developed and used previously in the United States to protect laboratorians routinely working with *F. tularensis*, no vaccine for tularemia is currently licensed for use in the United States.

### Outbreaks Related to War or Natural Disasters

Historically, risk for tularemia has increased with the social and ecologic disruption and poor sanitary conditions that result from war. A natural outbreak occurred during World War II when hundreds of thousands of persons developed tularemia in the former Soviet Union because of an increase in the number of infected rodents (*10*). Outbreaks of tularemia also were reported in the Karelia region of Finland during the 1941–1944 Continuation War and after the civil wars in Bosnia and Kosovo (*19*). The 1999–2000 outbreak in Kosovo involved >900 suspected cases, of which 327 were confirmed and spanned eastern, western, and central Kosovo. Investigation revealed the outbreak was likely related to a sharp, postwar increase in the number of rodents and subsequent contamination of food and water sources by rodent excrement and carcasses (*20*).

Natural disasters or other disruptions also can lead to outbreaks. A cluster of >130 oropharyngeal tularemia cases occurred in Turkey several years after a 1999 earthquake led to disruption of water infrastructure and construction of new settlements. Investigators determined the likely source of the outbreak was natural stream water that had been contaminated by infected rodents or other animals and used by local residents for drinking and cooking (*21*).

### *F. tularensis* as a Biologic Weapon

In 2012, *F. tularensis* was classified by the U.S. Federal Select Agent Program as a Tier 1 Select Agent, indicating the greatest risk for use during a bioterrorism attack based on its history of weaponization, stability in the environment, and an infectious dose as low as 10 organisms (*7*,*22*). The United States and the Soviet Union included the bacterium in their biologic weapon stockpiles during the 1950s and 1960s (*19*). These countries, as well as Japan and the United Kingdom, are known to have researched the bioweapon potential of *F. tularensis* (*10*). One documented U.S. study during 1966–1967, operation Red Cloud, involved mechanical dissemination of *F. tularensis* into an Alaskan forest to investigate distribution and decay rates (*23*). *F. tularensis* can be lyophilized and stored in a dried form and has successfully been engineered for resistance to multiple antibiotics, increasing concern for its use in a bioterrorism attack (*24*). Notably, former Soviet scientists have reported that *F. tularensis* strains resistant to antibiotics and vaccines were engineered by the Soviet Union as part of an offensive bioweapon program (*19*,*24*).

The World Health Organization assessed the potential impact of a bioterrorism attack and predicted that the release of 50 kg of aerosolized *F. tularensis* in a metropolitan area with a population of 5 million would result in approximately 250,000 incapacitated persons and 19,000 deaths under specific atmospheric and wind conditions. The effects of such an attack would likely last weeks to months because certain patients would experience prolonged illness or relapse attributable to sequestered bacteria and inadequate antimicrobial treatment. Furthermore, the release could result in new reservoirs of the bacteria in mammals and the environment, leading to subsequent outbreaks in animals and humans (*25*).

### Rationale for Guideline Development

To mitigate the effects of biologic, chemical, radiologic, or nuclear threats to the public, the U.S. government has committed substantial resources to research, develop, procure, and stockpile medical countermeasures (MCMs), including but not limited to antibiotics, vaccines, and antitoxins. The Strategic National Stockpile maintains MCMs for use during outbreaks and larger public health emergencies (*26*). Pursuant to the 21st Century Cures Act (*27*), CDC develops and maintains timely and accurate MCM use guidelines to treat or prevent threat-based diseases, including tularemia.

In 2001, guidelines for antimicrobial treatment and postexposure prophylaxis (PEP) of tularemia in the United States were published by the Working Group on Civilian Biodefense (*7*). These recommendations incorporated input from subject matter experts from CDC, clinical medicine, public health, and bioterrorism preparedness. Since then, additional animal data and human clinical data on effectiveness of various antimicrobials used to treat tularemia have become available (*4*,*28*,*29*), as have additional safety data regarding adverse effects of these drugs. In light of these developments, CDC has created updated clinical guidelines for tularemia using a systematic, evidence-based process that considered new evidence available since the previous guidelines were published (Box).

BOX **Summary of CDC recommendations for antimicrobial treatment and prophylaxis of tularemia, United States, 2025**

**Treatment recommendation for adults and children**

In persons with tularemia, CDC recommends first-line antimicrobial therapy with ciprofloxacin, levofloxacin, gentamicin, or doxycycline for adults and children aged ≥1 month and ciprofloxacin or gentamicin for neonates (aged ≤28 days).

- The choice between antimicrobials depends on severity of disease, availability of the drug, and shared decision-making, including discussion of the route of administration and side-effect profile.
- When intentional release of *F. tularensis* is suspected, dual antimicrobial therapy is recommended for initial treatment.
- When treatment initiation is delayed, ciprofloxacin, levofloxacin, or gentamicin is preferred over doxycycline.
- Treatment duration is 10 days for ciprofloxacin, levofloxacin, or gentamicin and 14–21 days for doxycycline.

**Treatment recommendation for pregnant women**

In pregnant women with tularemia, CDC recommends first-line antimicrobial management with ciprofloxacin, levofloxacin, or gentamicin.

- The choice between the three antimicrobials depends on severity of disease, availability of the drug, and shared decision-making including discussion of the route of administration and side-effect profile.
- When intentional release of *F. tularensis* is suspected, dual antimicrobial therapy is recommended for initial treatment.
- The treatment duration for ciprofloxacin, levofloxacin, and gentamicin is the same as for the nonpregnant adult population.

**Postexposure prophylaxis recommendation for adults (including pregnant women) and children**

For postexposure prophylaxis (PEP) for adults and children potentially exposed to *Francisella tularensis*, CDC recommends using ciprofloxacin, levofloxacin, or doxycycline for nonpregnant adults, children, and neonates and ciprofloxacin or levofloxacin for pregnant women.

- PEP should not be delayed if the preferred antimicrobial is not immediately available. Providers should begin prophylaxis with an available alternative antimicrobial until the preferred antimicrobial is available.
- PEP duration is 7 days for ciprofloxacin and levofloxacin and 10–14 days for doxycycline.

### Scope and Audience

The central purpose of these updated guidelines is to provide evidence-based PEP and treatment recommendations to U.S. clinicians, public health practitioners, and first responders. These recommendations can be used to manage *F. tularensis* exposures, individual tularemia cases, and outbreaks in the setting of naturally occurring disease or an intentional release of *F. tularensis*. These guidelines are not intended as a substitute for professional medical judgment or individual, patient-focused risk-benefit analyses. Although these guidelines contain emergency situation considerations, they do not include detailed crisis standards of care recommendations, defined by the Institute of Medicine as a substantial change in usual health care operations and the level of care possible to deliver during pervasive or catastrophic disaster (e.g., pandemic or hurricane) (*30*).

## Methods

These guidelines were created in accordance with established standards for the development of CDC evidence-based guidelines (*31*). The process included four major components: 1) systematic literature reviews, 2) analysis of U.S. surveillance data, 3) two topic sessions, and 4) one expert forum. Oversight and direction throughout the guideline development process was provided by a steering committee comprising CDC and Office of the Assistant Secretary for Preparedness and Response (ASPR) staff members with diverse expertise.

To ensure the best available evidence was used for guideline development, CDC’s Tularemia Clinical Guidelines Team conducted a systematic literature review of published cases of tularemia in humans who received antimicrobial treatment; the search strategy and review methods are detailed elsewhere (*4*). Case reports, case series, cohort studies, and controlled trials containing patient-level data, including antimicrobial treatment and outcome, were included. The resulting published review detailed 870 tularemia cases from 1993 to March 2023 described from generally lower-quality evidence sources; the majority of cases were from reports assessed to have a medium risk for bias (60% [n = 522]), 28.9% (n = 251) had a high risk for bias, and only 11.1% [n = 97] of the cases were from reports that received a low risk for bias assessment using the modified Newcastle-Ottawa evidence quality scale (*4*). An additional 263 tularemia cases in literature published during 1960–1992 also were summarized for discussion with experts, and a separate systematic review detailing tularemia cases in pregnant women identified and summarized 52 cases from 1930 to March 2023 (*32*). Both were similarly based on case reports and series (i.e., lower quality of evidence).

These guidelines also were informed by an analysis of U.S. surveillance data. Tularemia has been a nationally notifiable condition in the United States since 1927, with a hiatus during 1994–2000 (*33*). Individual case information reported to the National Notifiable Diseases Surveillance System was supplemented with case report forms when submitted by public health practitioners who conducted case investigations. CDC epidemiologists analyzed 1,153 cases that contained information on antimicrobial treatment and patient outcomes during 2006–2021 (*34*). A total of 166 U.S. cases of tularemia published in the scientific literature were included in the systematic literature review of tularemia (*4*); certain but not all of these cases also were reported via U.S. surveillance and thus included in both analyses. Existing antimicrobial safety data and tularemia treatment guidelines published by international agencies (*1*,*35*–*37*) also were evaluated during development of these guidelines.

In September 2022, CDC convened two topic sessions with approximately 65 participants, including CDC staff members, external subject matter experts, and Federal and local agency representatives. The first topic session focused on the treatment and prophylaxis of tularemia among adults and children, and the second session concentrated on neonates, breastfeeding infants, and pregnant or lactating women. During each topic session, participants reviewed data and provided individual expert input on clinical considerations and options for antimicrobial recommendations to inform CDC guidance. Input from these sessions was incorporated into CDC’s draft recommendations, with the goal of refining the recommendations after discussions with public health and clinical experts during the expert forum.

In February 2024, CDC convened an expert forum on the antibiotic treatment and prophylaxis of tularemia. Forum participants included approximately 85 clinical and public health experts, with representatives from the following Federal agencies: CDC, ASPR, Food and Drug Administration (FDA), National Institutes of Health (NIH), and Uniformed Services University of the Health Sciences. Clinical organization representatives from the American Academy of Pediatrics, Infectious Diseases Society of America, American Geriatrics Society, American College of Emergency Physicians, American College of Obstetricians and Gynecologists (ACOG), Society of Critical Care Medicine, and Society for Maternal-Fetal Medicine (SMFM) also were present. Representatives from the Association of State and Territorial Health Officials, Council of State and Territorial Epidemiologists, National Association of County and City Health Officials, Robert Koch Institute, and multiple state public health departments also participated. Finally, tularemia experts from domestic and international universities, clinics, and hospitals were in attendance to provide individual expertise and professional opinions.

During the expert forum, subject matter experts presented data from systematic literature reviews and U.S. surveillance reports. Published data from an NIH study regarding the efficacy of doxycycline and ciprofloxacin for treatment of tularemia in nonhuman primates were presented, as was information on the risks and adverse effects of potential antimicrobial treatments (*38*). The 2-day forum included breakout sessions on specific topics and special populations, including geriatric persons and persons with immunocompromise, neonates and lactating mothers, and pregnant women. Individual expert opinions on antimicrobial treatment and prophylaxis options were recorded and reviewed. CDC did not seek collective advice or attempt to achieve consensus among the forum attendees. The CDC Tularemia Clinical Guidelines Team assessed the available evidence, considered different perspectives and feedback shared in the expert forums, and used the Grading of Recommendations, Assessment, Development and Evaluation (GRADE) summary of findings and the Evidence to Decision (EtD) framework to ultimately formulate a recommendation based on the balance of benefits and harms and consensus among the CDC work group members.

Questionnaires were completed by all non-Federal staff, subject matter experts, and representatives who participated in guideline development to review and mitigate potential competing interests. Federal employees, including CDC staff members, are subject to the Standards of Ethical Conduct for Employees of the Executive Branch.*

The GRADE approach was applied in consultation with a GRADE specialist while developing these evidence-based guidelines. GRADE is a systematic, transparent, and widely used tool for assessing the quality of evidence and strength of recommendations of clinical guidelines (*39*). GRADE’s EtD framework was used to determine the certainty of evidence for key clinical outcomes related to antimicrobial treatment and prophylaxis of tularemia in adults, children, and pregnant women.

These recommendations include alternative and third-tier antimicrobial agents to provide multiple options for treatment and prophylaxis of tularemia if first-line antimicrobials are unavailable. Certain antimicrobials might not be readily available in the United States because of limited production or other factors; however, manufacturing surges, importation, or other mechanisms could be used to augment the domestic supply of specific antimicrobials when necessary.

## Recommendations

### Changes and Updates to Previous Recommendations

These guidelines (Tables 1, 2, 3, 4, 5, and 6) (Box) include the following notable changes compared with the recommendations published in 2001:

**TABLE 1:** Treatment of adults and children with tularemia — CDC recommendations for naturally acquired infections and bioterrorism response, United States, 2025

Population	Category	Antimicrobial class	Antimicrobial	Dosage	Duration (days)
**Adults aged ≥18 yrs**	First-line	Fluoroquinolone	Ciprofloxacin*	400 mg every 8 hrs IV or 750 mg every 12 hrs orally	10
			Levofloxacin*	750 mg every 24 hrs IV or orally	10
		Aminoglycoside	Gentamicin*	6 mg/kg every 24 hrs IV or IM^†^	10
		Tetracycline	Doxycycline	200 mg loading dose, then 100 mg every 12 hrs IV or orally	14–21
	Alternative	Fluoroquinolone	Moxifloxacin*^,§^	400 mg every 24 hrs IV or orally	10
			Ofloxacin*^,¶^	400 mg every 12 hrs orally	10
		Aminoglycoside	Amikacin*	15 mg/kg every 24 hrs IV or IM^†^	10
			Plazomicin*	15 mg/kg every 24 hrs IV^†^	10
			Tobramycin*	6 mg/kg every 24 hrs IV or IM^†^	10
		Tetracycline	Tetracycline	500 mg every 6 hrs orally	14–21
**Children and adolescents aged ≥1 mo to ≤17 yrs (unless otherwise noted)**	First-line	Fluoroquinolone	Ciprofloxacin*	10 mg/kg every 8–12 hrs IV (maximum 400 mg/dose) or 15 mg/kg every 8–12 hrs orally (maximum 500 mg/dose every 8 hrs or 750 mg/dose every 12 hrs)	10
			Levofloxacin*	Infants and children aged <5 yrs: 10 mg/kg every 12 hours IV or orally (maximum 375 mg/dose) Children and adolescents aged ≥5 yrs: 10 mg/kg every 24 hours IV or orally (maximum 750 mg/dose)	10
		Aminoglycoside	Gentamicin*	5–7.5 mg/kg every 24 hrs IV or IM^†^ (mg/kg dose determined by age**; upper range can be increased to 9.5 mg/kg based on age and AUC monitoring)	10
		Tetracycline	Doxycycline	4.4 mg/kg loading dose (maximum 200 mg), then 2.2 mg/kg every 12 hrs IV or orally (maximum 100 mg/dose)	14–21
	Alternative	Fluoroquinolone	Moxifloxacin*^,§^	Infants and children aged ≥3 mos to ≤23 mos: 6 mg/kg every 12 hrs IV or orally Children aged 2–5 yrs: 5 mg/kg every 12 hrs IV or orally Children aged 6–11 yrs: 4 mg/kg every 12 hrs IV or orally Children and adolescents aged 12 to ≤17 yrs: Body weight <45 kg: 4 mg/kg every 12 hrs IV or orally (maximum dose for all children weighing <45 kg: 200 mg/dose) Body weight ≥45 kg: 400 mg every 24 hrs IV or orally	10
			Ofloxacin*^,¶^	7.5 mg/kg every 12 hrs orally (maximum 400 mg/dose)	10
		Aminoglycoside	Amikacin*	15–20 mg/kg every 24 hrs IV or IM^†^	10
			Tobramycin*	5–7 mg/kg every 24 hrs IV or IM^†^	10
		Tetracycline	Tetracycline^††^	10 mg/kg every 6 hrs orally (maximum 500 mg/dose)	14–21
• Patients who are treated initially with an IV or IM antimicrobial can be transitioned to an oral regimen once they have defervesced and clinically improved. • Switching antimicrobial class is permissible, if needed. • Antimicrobials are listed alphabetically within each class. • For patients with severe infection, health care providers should consider treating initially with an aminoglycoside, if possible. Combination therapy with two classes of effective antimicrobials can also be used for severe tularemia (e.g., gentamicin plus ciprofloxacin or gentamicin plus doxycycline), although there is minimal evidence that initial treatment with two distinct classes of antimicrobials improves outcomes. Doxycycline monotherapy should not be used for patients with severe infection or substantial delays in treatment (>2 weeks). • Treatment recommendations for geriatric patients and those with immunocompromise do not differ from those for the general population. However, health care providers should recognize the potential for polypharmacy with resultant drug–drug interactions and adjust antimicrobials accordingly.					

**Abbreviations:** AUC = area under the curve (i.e., drug exposure over 24 hours); FDA = Food and Drug Administration; IM = intramuscular; IV = intravenous.

* Not approved by the FDA for treatment of tularemia. Ciprofloxacin, levofloxacin, and gentamicin have been used frequently off-label for the treatment of naturally occurring tularemia in humans. Large-scale distribution and use of these antimicrobials after a mass exposure event would be at the discretion of the FDA under an Emergency Use Authorization or other authority.

^†^ Extended-interval dosing. Monitor drug levels and renal function; extend interval further (beyond 24 hours) if indicated.

^§^ Moxifloxacin suspension for oral liquid administration is not available in the United States; however, hospitals and compounding retail pharmacies can use a published recipe to make a liquid suspension. Moxifloxacin is not FDA-approved for use in children and adolescents aged ≤17 years but has been used off-label (**Source:** Dixit A, Karandikar MV, Jones S, Nakamura MM. Safety and tolerability of moxifloxacin in children. J Pediatric Infect Dis Soc 2018;7:e92–101). For children and adolescents aged 12–17 years weighing ≥45 kg with risk factors for cardiac events, consider 200 mg twice daily to reduce risk for QT prolongation.

^¶^ Ofloxacin suspension for oral liquid administration is not available in the United States; however, hospitals and compounding retail pharmacies can use a published recipe to make a liquid suspension. Ofloxacin is not FDA-approved for use in children and adolescents aged ≤17 years but has been used off-label (**Source:** Garcia-Prats AJ, Draper HR, Thee S, et al. Pharmacokinetics and safety of ofloxacin in children with drug-resistant tuberculosis. Antimicrob Agents Chemother 2015;59:6073–9).

** Adjust dose as needed based on drug levels and renal function. Consult local guidelines. Certain references suggest 7.5 mg/kg/day IV or IM every 24 hours for patients aged 1 month to ≤10 years and 6 mg/kg/day IV or IM every 24 hours for patients aged >10 years (**Sources:** Bialkowski S, Staatz CE, Clark J, Lawson R, Hennig S. Gentamicin pharmacokinetics and monitoring in pediatric patients with febrile neutropenia. Ther Drug Monit 2016;38:693–8 and Hartman SJF, Orriëns LB, Zwaag SM, Poel T, de Hoop M, de Wildt SN. External validation of model-based dosing guidelines for vancomycin, gentamicin, and tobramycin in critically ill neonates and children: a pragmatic two-center study. Paediatr Drugs 2020;22:433–44).

^††^ Because of the risk for permanent tooth discoloration and tooth enamel hypoplasia, tetracycline should only be used for children aged <8 years when other options are unavailable.

**TABLE 2:** Postexposure prophylaxis for adults and children potentially exposed to *Francisella tularensis* — CDC recommendations for occupational exposures and bioterrorism response, United States, 2025

Population	Category	Antimicrobial class	Antimicrobial	Dosage	Duration (days)
**Adults aged ≥18 yrs**	First-line	Fluoroquinolone	Ciprofloxacin	500 mg every 12 hrs orally	7
			Levofloxacin	500 mg every 24 hrs orally	7
		Tetracycline	Doxycycline	100 mg every 12 hrs orally	10–14
	Alternative	Fluoroquinolone	Moxifloxacin*	400 mg every 24 hrs orally	7
			Ofloxacin^†^	400 mg every 12 hrs orally	7
		Tetracycline	Minocycline	100 mg every 12 hrs orally	10–14
			Tetracycline	500 mg every 6 hrs orally	10–14
		Macrolide	Azithromycin (for Type A and susceptible Type B biovars^§^)	500 mg every 24 hrs orally	10
**Children and adolescents aged ≥1 mo to ≤17 yrs (unless otherwise noted)**	First-line	Fluoroquinolone	Ciprofloxacin	15 mg/kg every 12 hrs orally (maximum 500 mg/dose)	7
			Levofloxacin	Infants and children aged <5 yrs: 8 mg/kg every 12 hours orally (maximum 250 mg/dose) Children aged ≥5 yrs and adolescents: 8 mg/kg every 24 hours orally (maximum 500 mg/dose)	7
		Tetracycline	Doxycycline	2.2 mg/kg every 12 hrs orally (maximum 100 mg/dose)	10–14
	Alternative	Fluoroquinolone	Moxifloxacin*	Infants and children aged ≥3 mos to ≤23 mos: 6 mg/kg every 12 hrs orally Children aged 2–5 yrs: 5 mg/kg every 12 hrs orally Children aged 6–11 yrs: 4 mg/kg every 12 hrs orally Children and adolescents aged 12 to ≤17 yrs: Body weight <45 kg: 4 mg/kg every 12 hrs orally (maximum dose for all children weighing <45 kg: 200 mg/dose) Body weight ≥45 kg: 400 mg every 24 hrs orally	7
			Ofloxacin^†^	7.5 mg/kg every 12 hrs orally (maximum 400 mg/dose)	7
		Tetracycline	Minocycline^¶^	2 mg/kg every 12 hrs orally (maximum 100 mg/dose)	10–14
			Tetracycline^¶^	10 mg/kg every 6 hrs orally (maximum 500 mg/dose)	10–14
		Macrolide	Azithromycin (for Type A and susceptible Type B biovars^§^)	Body weight <45 kg: 10 mg/kg every 24 hrs orally Body weight ≥45 kg: 500 mg every 24 hrs orally	10
• Antimicrobials are listed alphabetically within each class. • Prophylaxis recommendations for geriatric patients and those with immunocompromise do not differ from those for the general population. However, health care providers should recognize the potential for polypharmacy with resultant drug–drug interactions and adjust antimicrobials accordingly. • Note: The antibiotics listed in this table are not approved by the FDA for prophylaxis of tularemia. Ciprofloxacin has been used frequently off-label for prophylaxis of naturally occurring tularemia in humans. Large-scale distribution and use of these antimicrobials after a mass exposure event would be at the discretion of the FDA under an Emergency Use Authorization or other authority.					

**Abbreviations:** FDA = Food and Drug Administration; PEP = postexposure prophylaxis.

* Moxifloxacin suspension for oral liquid administration is not available in the United States; however, hospitals and compounding retail pharmacies can use a published recipe to make a liquid suspension. Moxifloxacin is not FDA-approved for use in children and adolescents aged ≤17 years but has been used off-label (**Source:** Dixit A, Karandikar MV, Jones S, Nakamura MM. Safety and tolerability of moxifloxacin in children. J Pediatric Infect Dis Soc 2018;7:e92–101). For children and adolescents aged 12–17 years weighing ≥45 kg with risk factors for cardiac events, consider 200 mg twice daily to reduce risk for QT prolongation.

^†^ Ofloxacin suspension for oral liquid administration is not available in the United States; however, hospitals and compounding retail pharmacies can use a published recipe to make a liquid suspension. Ofloxacin is not FDA-approved for use in children and adolescents aged ≤17 years but has been used off-label (**Source:** Garcia-Prats AJ, Draper HR, Thee S, et al. Pharmacokinetics and safety of ofloxacin in children with drug-resistant tuberculosis. Antimicrob Agents Chemother 2015;59:6073–9).

^§^ *Francisella tularensis* subspecies *tularensis* (Type A) is limited to North America and susceptible to macrolides. *F. tularensis* subspecies *holarctica* (Type B) biovar I strains (in North America and Western Europe) and biovar *japonica* strains (primarily found in Japan) are also susceptible to macrolides. *F. tularensis* subspecies *holarctica* biovar II strains found in Eastern Europe and Asia are inherently resistant to macrolides. Thus, azithromycin can be used if PEP is indicated after natural occupational exposures in the United States (e.g., contact with an infected animal). In the wake of an intentional release, azithromycin can be used for PEP initially if needed. If additional information identifies a resistant strain, patients should be switched to another antimicrobial.

^¶^ Because of the risk for permanent tooth discoloration and tooth enamel hypoplasia, tetracycline and minocycline should only be used for children aged <8 years when other options are unavailable.

**TABLE 3:** Treatment of pregnant women with tularemia — CDC recommendations for naturally acquired infections and bioterrorism response, United States, 2025

Category	Antimicrobial class	Antimicrobial	Dosage	Duration (days)
**First-line**	Fluoroquinolone	Ciprofloxacin*	400 mg every 8 hrs IV or 750 mg every 12 hrs orally^†^	10
		Levofloxacin*	750 mg every 24 hrs IV or orally	10
	Aminoglycoside	Gentamicin*	6 mg/kg every 24 hrs IV or IM^§^	10
**Alternative**	Fluoroquinolone	Moxifloxacin*^,¶^	400 mg every 24 hrs IV or orally	10
		Ofloxacin*^,¶^	400 mg every 12 hrs IV or orally	10
	Aminoglycoside	Amikacin*	15 mg/kg every 24 hrs IV or IM^§^	10
		Tobramycin*	6 mg/kg every 24 hrs IV or IM^§^	10
	Tetracycline	Doxycycline	200 mg loading dose, then 100 mg every 12 hrs IV or orally	14–21
• Patients who are treated initially with an IV or IM antimicrobial can be transitioned to an oral regimen once they have defervesced and clinically improved. • Switching antimicrobial class is permissible, if needed. • Antimicrobials are listed alphabetically within each class. • For pregnant patients with severe infection, health care providers should consider treating initially with an aminoglycoside if possible. Combination therapy with two classes of effective antimicrobials can also be used for severe tularemia (e.g., gentamicin plus ciprofloxacin), although there is minimal evidence that initial treatment with two distinct classes of antimicrobials improves outcomes. Doxycycline monotherapy should not be used for patients with severe infection or substantial delays in treatment (>2 weeks).				

**Abbreviations:** FDA = Food and Drug Administration; IM = intramuscular; IV = intravenous.

* Not approved by the FDA for treatment of tularemia. Ciprofloxacin, levofloxacin, and gentamicin have been used frequently off-label for the treatment of naturally occurring tularemia in humans. Large-scale distribution and use of these antimicrobials after a mass exposure event would be at the discretion of the FDA under an Emergency Use Authorization or other authority.

^†^ For patients in the third trimester of pregnancy, consider 500 mg every 8 hours.

^§^ Extended-interval dosing. Monitor drug levels and extend interval further (beyond 24 hours) if indicated. However, for pregnant patients, certain experts recommend conventional dosing of aminoglycosides rather than extended-interval dosing. Consult local guidelines.

^¶^ Moxifloxacin and ofloxacin suspensions for oral liquid administration are not available in the United States; however, hospitals and compounding retail pharmacies can use a published recipe to make a liquid suspension.

**TABLE 4:** Postexposure prophylaxis for pregnant women potentially exposed to *Francisella tularensis* — CDC recommendations for occupational exposures and bioterrorism response, United States, 2025

Category	Antimicrobial class	Antimicrobial	Dosage	Duration (days)
**First-line**	Fluoroquinolone	Ciprofloxacin	500 mg every 12 hrs orally	7
		Levofloxacin	500 mg every 24 hrs orally	7
**Alternative**	Tetracycline	Doxycycline	100 mg every 12 hrs orally	10–14
	Fluoroquinolone	Moxifloxacin*	400 mg every 24 hrs orally	7
		Ofloxacin*	400 mg every 12 hrs orally	7
	Macrolide	Azithromycin (for Type A and susceptible Type B biovars^†^)	500 mg every 24 hrs orally	10
• Antimicrobials are listed alphabetically within the fluoroquinolone class. • Note: The antibiotics listed in this table are not approved by the FDA for prophylaxis of tularemia. Ciprofloxacin has been used frequently off-label for prophylaxis of naturally occurring tularemia in humans. Large-scale distribution and use of these antimicrobials after a mass exposure event would be at the discretion of the FDA under an Emergency Use Authorization or other authority.				

**Abbreviations:** FDA = Food and Drug Administration; PEP = postexposure prophylaxis.

* Moxifloxacin and ofloxacin suspensions for oral liquid administration are not available in the United States; however, hospitals and compounding retail pharmacies can use a published recipe to make a liquid suspension.

^†^ *Francisella tularensis* subspecies *tularensis* (Type A) is limited to North America and susceptible to macrolides. *F. tularensis* subspecies *holarctica* (Type B) biovar I strains (in North America and Western Europe) and biovar *japonica* strains (primarily found in Japan) are also susceptible to macrolides. *F. tularensis* subspecies *holarctica* biovar II strains found in Eastern Europe and Asia are inherently resistant to macrolides. Thus, azithromycin can be used if PEP is indicated after natural occupational exposures in the United States (e.g., contact with an infected animal). In the wake of an intentional release, azithromycin can be used for PEP initially if needed. If additional information identifies a resistant strain, patients should be switched to another antimicrobial.

**TABLE 5:** Treatment of neonates aged ≤28 days with tularemia — CDC recommendations for naturally acquired infections and bioterrorism response, United States, 2025

Category	Antimicrobial class	Antimicrobial	Dosage	Duration (days)
**First-line**	Fluoroquinolone	Ciprofloxacin*	10 mg/kg every 8 or 12 hrs IV	10
	Aminoglycoside	Gentamicin*	30–34 weeks’ gestational age: Postnatal age ≤10 days: 5 mg/kg every 36 hrs IV or IM^†^ Postnatal age >10 days: 5 mg/kg every 24 hrs IV or IM^†^ ≥35 weeks’ gestational age: 5 mg/kg every 24 hrs IV or IM^†^	10
**Alternative**	Fluoroquinolone	Levofloxacin*	10 mg/kg every 12 hrs IV	10
	Aminoglycoside	Amikacin*	30–34 weeks’ gestational age: Postnatal age ≤10 days: 15 mg/kg every 36 hrs IV or IM^†^ Postnatal age >10 days: 15 mg/kg every 24 hrs IV or IM^†^ ≥35 weeks’ gestational age: Postnatal age ≤7 days: 15 mg/kg every 24 hrs IV or IM^†^ Postnatal age >7 days: 18 mg/kg every 24 hrs IV or IM^†^	10
		Tobramycin*	30–34 weeks’ gestational age: Postnatal age ≤10 days: 5 mg/kg every 36 hrs IV or IM^†^ Postnatal age >10 days: 5 mg/kg every 24 hrs IV or IM^†^ ≥35 weeks’ gestational age: 5 mg/kg every 24 hrs IV or IM^†^	10
	Tetracycline	Doxycycline	4.4 mg/kg loading dose, then 2.2 mg/kg every 12 hrs IV	14–21
• Neonates who are treated initially with an IV or IM antimicrobial can be transitioned to an oral regimen once they have defervesced and clinically improved. • Switching antimicrobial class is permissible, if needed. • Antimicrobials are listed alphabetically within the aminoglycoside class. • For neonates with severe infection, health care providers should consider treating initially with an aminoglycoside if possible. Combination therapy with two classes of effective antimicrobials can also be used for severe tularemia (e.g., gentamicin plus ciprofloxacin or gentamicin plus doxycycline), although there is minimal evidence that initial treatment with two distinct classes of antimicrobials improves outcomes. Doxycycline monotherapy should not be used for neonates with severe infection or substantial delays in treatment (>2 weeks).				

**Abbreviations:** FDA = Food and Drug Administration; IM = intramuscular; IV = intravenous.

* Not approved by the FDA for treatment of tularemia. Ciprofloxacin, levofloxacin, and gentamicin have been used frequently off-label for the treatment of naturally occurring tularemia in humans. Large-scale distribution and use of these antimicrobials after a mass exposure event would be at the discretion of the FDA under an Emergency Use Authorization or other authority.

^†^ Extended-interval dosing. Monitor drug levels and renal function; adjust dose and interval if necessary. Aminoglycoside dosing information for neonates <30 weeks’ gestational age can be found in the 2024–2027 Red Book (**Source:** American Academy of Pediatrics. Red Book: 2024–2027 report of the Committee on Infectious Diseases, 33rd edn. Kimberlin DW, Banerjee R, Barnett ED, Lynfield F, Sawyer MH, eds. Itasca, IL: American Academy of Pediatrics; 2024).

**TABLE 6:** Postexposure prophylaxis of neonates aged ≤28 days potentially exposed to *Francisella tularensis* — CDC recommendations for bioterrorism response, United States, 2025

Category	Antimicrobial class	Antimicrobial	Dosage	Duration (days)
**First-line**	Fluoroquinolone	Ciprofloxacin	12.5 mg/kg every 12 hrs orally	7
		Levofloxacin	10 mg/kg every 12 hrs orally	7
	Tetracycline	Doxycycline	2.2 mg/kg every 12 hrs orally	10–14
**Alternative**	Fluoroquinolone	Ofloxacin*	7.5 mg/kg every 12 hrs orally	7
• Antimicrobials are listed alphabetically within the fluoroquinolone class. • Note: The antibiotics listed in this table are not approved by the FDA for prophylaxis of tularemia. Ciprofloxacin has been used frequently off-label for prophylaxis of naturally occurring tularemia in humans. Large-scale distribution and use of these antimicrobials after a mass exposure event would be at the discretion of the FDA under an Emergency Use Authorization or other authority.				

**Abbreviation:** FDA = Food and Drug Administration.

* Ofloxacin suspension for oral liquid administration is not available in the United States; however, hospitals and compounding retail pharmacies can use a published recipe to make a liquid suspension. Ofloxacin is not FDA-approved for use in children and adolescents aged ≤17 years but has been used off-label (**Source:** Garcia-Prats AJ, Draper HR, Thee S, et al. Pharmacokinetics and safety of ofloxacin in children with drug-resistant tuberculosis. Antimicrob Agents Chemother 2015;59:6073–9).

1. Recommendations have been modified to align with two overarching categories: treatment and prophylaxis (pre- and postexposure). This approach replaces the previous paradigm, which divided recommendations into a contained casualty scenario (entirely intravenous [IV] or intramuscular [IM] treatment) versus a mass casualty scenario (all oral antimicrobials for either treatment or prophylaxis).
2. If intentional release of *F. tularensis* is confirmed or strongly suspected, health care providers are encouraged to treat symptomatic patients with suspected infection with two distinct classes of antimicrobial drugs, at least one of which is considered first-line, until antimicrobial susceptibility patterns are known. This treatment regimen increases the likelihood that at least one effective drug is administered.
3. Ciprofloxacin and doxycycline have been shifted from alternative choices for contained casualty treatment scenarios to first-line treatment options. Moreover, levofloxacin has been added as a first-line drug for treatment and prophylaxis. The supporting data and rationale for these changes are summarized in this report.
4. In addition to first-line and alternative options listed in the tables for treatment and PEP, third-tier options are described. Third-tier options can be used when other antimicrobials are unavailable or for patients with contraindications to first-line and alternative drugs because of allergies or other concerns.
5. Streptomycin has been relegated to a third-tier treatment option. Although streptomycin is highly effective for treatment of tularemia, it carries greater risk for certain adverse events compared with gentamicin and has limited availability in the United States. Moreover, the infrequency of streptomycin use in the United States could lead to greater risk for administration errors because health care providers are not accustomed to preparing and administering this drug.
6. Azithromycin has been added as an alternative option for PEP and as a third-tier option for treatment. Macrolides have demonstrated in vitro and in vivo efficacy against *F. tularensis *subspecies *tularensis* (Type A) and certain strains of *F. tularensis* subspecies *holarctica* (Type B); however, Eastern European Type B biovar II strains have inherent resistance against macrolides (*18*,*40*).
7. Chloramphenicol has been downgraded to a third-tier option for treatment rather than an alternative because of the risk for serious adverse events (e.g., aplastic anemia and neonatal cyanosis or gray syndrome). This medication can be used in unique situations when other treatment options are limited, based on a risk-benefit assessment.
8. Rifampin has been added as a third-tier option for prophylaxis. Rifamycins have demonstrated in vitro activity against *F. tularensis*; however, because of concerns for induced resistance, their use has been limited to combination therapy in certain clinical situations. During a response to a large-scale event, rifampin is a viable option for prophylaxis when supplies of first-line agents are unavailable.
9. Within-class antimicrobial alternatives have been added to treatment and prophylaxis recommendations. For example, moxifloxacin and ofloxacin are listed as alternative fluoroquinolone options and amikacin, tobramycin, and plazomicin are included as alternative aminoglycoside options. These additions are based in part on in vitro and animal data, when available, plus extrapolation from the evidence base for the first-line agents ciprofloxacin and gentamicin. These expanded treatment and prophylaxis options increase the repertoire of available antimicrobial drugs, which could be critical to meet surge capacity after a large-scale event.
10. Recommendations have been added for other special populations, including neonates, breastfeeding infants, lactating mothers, and geriatric patients.

### Evidence Review and Recommendations for Antimicrobial Treatment of Tularemia in Adults and Children

Recommended first-line and alternative antimicrobial agents for treatment of tularemia in adults and children are provided (Table 1). This section summarizes the evidence for use of specific antimicrobials and drug classes. Additional details on the evidence base are available (SupplementaryMaterial).

Streptomycin, doxycycline, tetracycline, and minocycline are currently the only antimicrobial agents with an FDA-approved indication for treatment of tularemia; no antimicrobials have an FDA-approved indication specifically for prophylaxis of tularemia. However, multiple additional antimicrobials, including ciprofloxacin and gentamicin, have been used off-label for treatment and prophylaxis of tularemia in children and adults. Off-label prescribing is a common medical practice, especially for rare diseases when FDA-approved drugs are lacking or inadequate. Seeking FDA approval for specific indications requires meeting regulatory requirements such as clinical trials that demonstrate efficacy and safety. This process is challenging even for antimicrobials with longstanding off-label clinical use for the disease, attributable to low incidence of disease, limited investment and market potential, and high research cost with lack of profitable commercial viability. A cynomolgus macaque animal model has been formally recognized for tularemia research under FDA’s Animal Model Qualification Program, which could potentially pave the way for additional FDA approvals for tularemia antibiotic treatment and prophylaxis in the future (*41*).

#### Fluoroquinolones

When the previous tularemia guidelines were published in 2001 (*7*), clinical evidence on effectiveness of fluoroquinolones to treat tularemia was limited. Since then, health care provider experiences and published research have expanded the knowledge base on the effectiveness of fluoroquinolones for treatment of tularemia.

**Ciprofloxacin.** Although ciprofloxacin is not currently FDA-approved for treatment of tularemia, it is considered highly effective based on in vitro and animal studies. Years of clinical experience and published case data also support ciprofloxacin as a first-line agent for tularemia treatment. The standard adult dose for ciprofloxacin is 400 mg every 8 hours IV or 750 mg every 12 hours orally, or for children, 10 mg/kg every 8–12 hours IV (maximum 400 mg/dose) or 15 mg/kg every 8–12 hours orally (maximum 500 mg/dose every 8 hours or 750 mg/dose every 12 hours).

In vitro studies have demonstrated that *F. tularensis* is susceptible to fluoroquinolones. Ciprofloxacin has been regularly found to have minimum inhibitory concentration (MIC) ranges of 0.015–0.12 *µ*g/mL for MIC50 and 0.016–0.25 *µ*g/mL for MIC90, with few differences between Type A and Type B strains. Animal studies also support the use of ciprofloxacin for tularemia treatment. Three mouse studies that directly compared ciprofloxacin with doxycycline for treatment of tularemia found similar or slightly better outcomes in the ciprofloxacin treatment groups (*42*–*44*). A direct comparison of ciprofloxacin versus doxycycline was also conducted in cynomolgus macaques after inhalational challenge of *F. tularensis*. Both antimicrobials were highly effective for treatment of symptomatic infection; however, rates of survival and tissue bacteria clearance were slightly greater among the ciprofloxacin-treated macaques (*38*).

During a 1998 outbreak in Sweden, 41 of 43 patients with ulceroglandular, typhoidal, or pneumonic tularemia were treated successfully with oral ciprofloxacin. Of the two patients who experienced complications, one had a treatment delay of 3 weeks and later developed a lymph node abscess, whereas the second had typhoidal and pneumonic disease that responded initially to ciprofloxacin but relapsed 7 days after treatment ended (*45*). Ciprofloxacin is also effective in treating children, as demonstrated in a study of 12 children aged ≤10 years with ulceroglandular tularemia who recovered without complications after 10–14 days of oral ciprofloxacin. Although defervescence occurred within 4 days of treatment, ciprofloxacin was discontinued in two (16.7%) children on days 3 and 7 of treatment because of development of a rash (*29*). Among children with tularemia in Switzerland, ciprofloxacin and doxycycline were used successfully for 15 and five patients, respectively. The authors did not detect a differential treatment response between patients treated with ciprofloxacin versus doxycycline, although small case numbers in these groups and treatment delays limited comparisons. No patients required additional therapy or surgery after receiving a course of ciprofloxacin or doxycycline (*46*).

**Levofloxacin.** Although ciprofloxacin has been the most commonly used fluoroquinolone for treating tularemia, existing animal data and clinical experience suggest that other antibiotics within this class are equally effective. Specifically, levofloxacin has emerged as a valid alternative to ciprofloxacin. Levofloxacin has the advantage of broad coverage for bacterial respiratory pathogens; thus, it can be particularly useful when tularemia is part of the differential diagnosis for patients with pneumonia but has not yet been confirmed. The standard dose for levofloxacin is 750 mg every 24 hours IV or orally for adults, or for children, 10 mg/kg IV or orally.

In vitro studies of levofloxacin have demonstrated a MIC50 range of 0.012–0.06 *µ*g/mL and a MIC90 range of 0.012–0.125 *µ*g/mL (*18*,*40*,*47*–*52*). In a mouse study, levofloxacin was 100% effective when administered 24 hours after inhalational exposure to the *F. tularensis* subspecies *tularensis* (Type A) Schu S4 strain, regardless of the dose. In addition, levofloxacin was effective when administration was delayed, with 100% survival among mice that received levofloxacin 48 or 72 hours post-exposure. Survival decreased to 80% when treatment began 96 hours post-exposure. Notably, mice successfully treated with levofloxacin remained healthy after rechallenge with Schu S4 (*53*).

Levofloxacin monotherapy has been used successfully to treat patients with tularemia, including two acutely ill patients with substantial immunocompromise (*54*). A systematic literature review of published individual tularemia cases worldwide found a higher CFR (7.7% [n = two of 26]) among patients who received levofloxacin; however, small case numbers limit interpretation of this finding (*4*). One patient who died was acutely ill upon hospital admission and received doxycycline in addition to levofloxacin, then subsequently ciprofloxacin and gentamicin (*55*). The other patient was not treated with levofloxacin until he was transferred to intensive care, then died the next day after massive gastrointestinal hemorrhage (*56*). Thus, these two fatal cases did not appear to be clear examples of levofloxacin failure.

**Moxifloxacin.** Moxifloxacin has also been demonstrated to be effective in mouse studies of PEP (*57*,*58*). Other fluoroquinolones, including ofloxacin, trovafloxacin, grepafloxacin, norfloxacin, pefloxacin, and cinoxacin, have demonstrated in vitro effectiveness against *F. tularensis*; however, further research is needed to assess in vivo efficacy (*59*,*60*).

#### Aminoglycosides

Aminoglycosides have been the preferred treatment for tularemia since streptomycin was first used to treat the disease in 1946. For patients with hemodynamic instability, end-organ dysfunction, need for respiratory support, or other signs of sepsis or severe illness, initial treatment with an aminoglycoside remains the preferred choice based on the greater clinical experience and published evidence demonstrating efficacy. However, aminoglycosides require parenteral administration and drug level monitoring, which might unnecessarily complicate management of patients with milder disease, who might be managed with oral agents.

**Gentamicin.** Gentamicin is currently considered the most effective and practical aminoglycoside for treatment of tularemia and has largely supplanted streptomycin because of the latter’s higher risk for certain adverse effects (e.g., irreversible hearing loss) (*61*,*62*) and limited availability in the United States. Gentamicin is recommended as a first-line agent for tularemia because of its low MIC, demonstrated efficacy in controlled studies of mice and nonhuman primates (*63*–*66*), and long record of successful use for patients with tularemia. Extended-interval dosing^†^ is 6 mg/kg every 24 hours IV or IM for adults and 5–7.5 mg/kg every 24 hours IV or IM for children for 10 days; the MIC ranges of gentamicin against *F. tularensis* are 0.06–0.25 *µ*g/mL for MIC50 and 0.06–1.0 *µ*g/mL for MIC90. Drug levels and renal function should be monitored during at least the initial days of therapy.

Evidence for the effectiveness of gentamicin for treatment of tularemia in humans has been supported by numerous observations and case series. A description of 10 patients treated with gentamicin reported a 100% survival rate without relapse. These cases included pulmonary, pericardial, typhoidal, and ulceroglandular manifestations, as well as a patient with severe pneumonia and several with renal impairment when therapy was initiated (*63*). A systematic literature review of cases before 1994 found that among 36 patients treated with gentamicin, 31 (86%) were successfully cured, two experienced relapse, two deteriorated, and one died. Thirteen of the successfully treated patients received only gentamicin. Two of the patients who experienced treatment failure with gentamicin had delays in initiation of therapy (including the patient who died), and one patient who relapsed had received <6 days of gentamicin therapy. In comparison, in the same review, 44 of 50 patients (88%) who received doxycycline recovered and six experienced relapses. Among 224 patients treated with streptomycin, 217 (97%) recovered, six died, and one deteriorated (*67*).

A case series published in 2019 described four patients with pneumonic tularemia who were successfully treated with gentamicin using extended-interval dosing. However, two of the patients also received ciprofloxacin as part of their treatment regimen (*68*). A more recent systematic review of published cases from 1993 to 2023 indicated a high success rate for treatment with gentamicin. When compared with streptomycin, patients who received gentamicin experienced a lower rate of lymph node complications, including need for aspiration, excision, and rupture, and a similar rate of thoracentesis or pleural effusion drainage. Intubation or respiratory support and fever recurrence were more common with gentamicin compared with streptomycin (6.4% versus 1.2% and 4.6% versus 1.8%, respectively), although treatment bias and small case numbers limit interpretation of these data. Notably, no deaths occurred among the 11 patients treated with gentamicin monotherapy for pneumonic tularemia, the most severe form of illness (*4*). Other aminoglycosides, namely amikacin, tobramycin, and plazomicin, can be considered as alternative treatment options if gentamicin is contraindicated and first-line antimicrobial drugs are not available.

**Amikacin.** Extended-interval dosing for amikacin is 15 mg/kg every 24 hours IV or IM for adults or 15–20 mg/kg every 24 hours IV or IM for children. Although human clinical data are not available for treatment of tularemia with amikacin specifically, in vitro studies indicate robust activity against *F. tularensis*, with a MIC50 of 1.0 *µ*g/mL and MIC90 of 2.0 *µ*g/mL (*47*,*69*). An animal study found that survival rates of mice treated with amikacin were slightly higher than mice treated with streptomycin after exposure to various strains of *F. tularensis* (*70*). When a streptomycin-resistant strain of *F. tularensis* was tested specifically, amikacin retained its efficacy and 10 of 10 mice survived, whereas zero of 10 streptomycin-treated mice survived (*70*).

**Tobramycin.** Extended-interval dosing for tobramycin is 6 mg/kg every 24 hours IV or IM for adults or 5–7 mg/kg every 24 hours IV or IM for children. In vitro studies of tobramycin found MIC50 ranges of 0.12–1.0 *µ*g/mL and MIC90 of 0.2–1.5 *µ*g/mL (*47*–*49*). Clinical reports of oculoglandular tularemia indicate tobramycin can be useful as a topical agent when prescribed with streptomycin (*71*). However, reviews of the literature indicated that two of six (33%) severely ill patients died when treated with tobramycin, with one patient lost to follow-up (*67*,*69*).

**Plazomicin.** In vitro and in vivo studies indicate that plazomicin is potentially effective for treating tularemia (*64*); the dose for adults is 15 mg/kg every 24 hours IV; the drug is not recommended for children because no published data exist on its use and dosage in the pediatric population. Plazomicin has a MIC50 of 0.5 *µ*g/mL and MIC90 of 1.0 *µ*g/mL (*72*,*73*). A study that challenged mice with the *F. tularensis* subspecies *tularensis* (Type A) Schu S4 strain indicated a favorable outcome with plazomicin treatment, with 90% survival 35 days after treatment for 7 days and 100% survival when treatment was extended to 10 or 14 days (*73*,*74*).

**Streptomycin.** Streptomycin should be considered for treatment of tularemia only when first-line and alternative antimicrobial drugs have been exhausted. Logistical barriers in procuring and administering streptomycin might exist because of its limited availability and minimal experience of U.S. prescribers, pharmacists, and nurses with ordering and preparing it. MIC ranges are 0.5–4.0 *µ*g/mL for MIC50 and 0.25–6.0 *µ*g/mL for MIC90 (*18*,*40*,*47*–*51*). The dose is 1 g IV or IM twice daily (IV use is off-label) for adults and 15 mg/kg IV or IM twice daily for children (maximum dose 2 g/day). Recommended treatment duration is 10 days.

#### Tetracyclines

Tetracycline class antimicrobials have been used for treatment of tularemia since the 1950s. Tetracycline was the oral antimicrobial of choice for many decades until doxycycline was developed and demonstrated to be effective for tularemia.

**Doxycycline.** Because of its long history of use for treatment of human tularemia, robust in vitro and animal data, and effectiveness observed with early treatment, evidence supports the use of doxycycline as a first-line agent for treatment of tularemia in most situations, even though there are no controlled studies of treatment in humans. Recommended dosing is 200 mg loading then 100 mg every 12 hours IV or orally for adults or 4.4 mg/kg loading (maximum 200 mg) then 2.2 mg/kg (maximum 100 mg) every 12 hours IV or orally for children. Treatment duration is 14–21 days.

*F. tularensis* is consistently susceptible to doxycycline based on in vitro studies, with a MIC50 range of 0.12–1.0 *µ*g/mL and a MIC90 range of 0.25–4.0 *µ*g/mL (*18*,*50*,*51*). Additional details and a summary of the EtD framework are available (SupplementaryMaterial).

Animal studies have demonstrated that doxycycline is generally effective for treatment and prophylaxis of tularemia. At least three published mouse studies directly compared doxycycline treatment with ciprofloxacin (*42*–*44*). One study found that 100% of mice infected via airway (intranasal) exposure with *F. tularensis* live vaccine strain (LVS) survived when treated with ciprofloxacin or doxycycline, even when treatment was delayed to 72 hours postinfection (*42*). In another study, all mice inoculated with a lethal dose of LVS survived after treatment with either ciprofloxacin or doxycycline 24–72 hours postinfection. For the group administered ciprofloxacin, bacteria were cleared more quickly from organs and the mice experienced less dramatic weight loss during infection (*43*). Another study found similar survival and relapse rates for mice infected intraperitoneally with *F. tularensis* Schu S4 then treated with either ciprofloxacin or doxycycline (40 mg/kg twice daily) 24 hours postinfection (*44*).

In other studies, survival rates of mice treated with doxycycline were slightly lower than for ciprofloxacin, particularly in situations of treatment delay. For example, when mice were infected with Schu S4 instead of LVS, survival rates for treatment administered 24, 48, or 72 hours postinfection were 100%, 100%, and 70% for ciprofloxacin but 90%, 30%, and 0% for doxycycline, respectively (*42*). Among the mice treated 72 hours postinfection, 30% of the ciprofloxacin-treated group and 100% of the doxycycline-treated group had detectable bacteria after cessation of treatment (*42*).

Doxycycline was highly effective in primate studies, with similar survival rates observed in the ciprofloxacin- and doxycycline-treated groups. In a controlled study of *F. tularensis* inhalational challenge in cynomolgus macaques, 20 of 20 animals treated with ciprofloxacin and 19 of 20 animals treated with doxycycline survived. Among the 10 macaques treated with doxycycline 48 hours postinfection, *F. tularensis* was detected by plate culture in the liver (one animal), spleen (two animals), bronchial lymph node (three animals) and mesenteric lymph node (one animal) but not in the lung, brain, kidney, or other lymph nodes. Tissues from the ciprofloxacin-treated groups were sterile upon euthanasia. The animal that did not survive was treated with doxycycline 24 hours after onset of fever and initially experienced clinical recovery but was euthanized because of signs of severe illness on study day 17, before the cessation of treatment. On examination, “disease pathology was not consistent with tularemia as a cause of death … and … a cause of death could not be established, based on histopathology” (*38*). Two lymph node specimens from this animal were positive for *F. tularensis* by plate culture, albeit below the lower level of quantitation. Test results for the remaining tissues were negative for *F. tularensis* by plate culture (*38*).

**Tetracycline.** Although doxycycline has replaced tetracycline as first-line treatment of tularemia in adults, tetracycline remains an acceptable second-line agent. The recommended dose of tetracycline is 500 mg every 6 hours orally for adults and 10 mg/kg every 6 hours orally (maximum 500 mg/dose) for children aged ≥8 years. Investigations of tetracycline performed in U.S. human volunteers during the 1950s–1960s found that among 44 volunteers exposed to airborne *F. tularensis* Schu S4 and treated 1–5 days after fever onset, all experienced complete recovery without relapse when treated with tetracycline 2 g/day for ≥14 days. Eight of these patients (18.2%) experienced a minor adverse event (e.g., transient increase in body temperature) after termination of tetracycline; however, symptoms spontaneously resolved in all patients. Volunteers treated with a lower dose (1 g/day) or shorter duration (10 days) of tetracycline had relapse rates of 25% and 45.5%, respectively (*64*,*75*) and required subsequent treatment with streptomycin.

#### Additional Antimicrobial Classes

**Macrolides.** The effectiveness of macrolides against *F. tularensis* is highly dependent on the strain. Type A strains are uniformly sensitive to azithromycin and erythromycin, with MICs in the range of 0.064–8.0 *µ*g/mL (*50*–*52*,*76*). Type B strains, typically divided into three biovars, exhibit differential susceptibility by biovar. Biovar I is inherently susceptible to macrolides, with MIC ranges of 0.064–2.0 *µ*g/mL for azithromycin and 0.125–8.0 *µ*g/mL for erythromycin (*52*,*76*). Biovar II is naturally resistant to macrolides, with demonstrated MICs of >256 *µ*g/mL for both azithromycin and erythromycin (*18*,*52*). Type B biovar II strains also have demonstrated resistance to roxithromycin and clarithromycin (*40*,*49*,*50*,*76*). Type B *japonica* strains have a MIC90 range of 0.094–1.5 *µ*g/mL for erythromycin and are considered generally susceptible to macrolides (*40*).

The efficacy of macrolides as treatment for human tularemia has not been examined in controlled clinical trials. Nevertheless, azithromycin has been used as a first-line treatment for pregnant women with tularemia because of the known safety profile and experience with this antimicrobial within this population.

Azithromycin has been used successfully to treat three pregnant women with glandular, ulceroglandular, and oropharyngeal tularemia when treatment began at 6–20 weeks’ gestation. Treatment duration was unknown for one patient and 4–6 weeks for two patients. In all cases, the mothers recovered, and their babies were born without complications and in good health (*77*–*79*).

**Chloramphenicol.** Chloramphenicol has been used for many years to treat tularemia, although certain publications have reported lower efficacy in humans compared with streptomycin (*2*,*80*). Chloramphenicol is associated with serious risks for adverse events to patients, including aplastic anemia in patients of all ages and neonatal or infant cyanosis and vasomotor collapse (gray syndrome.). Risks also exist to pharmacy and nursing staff during preparation and administration of the drug. Among pregnant women, the risk for congenital malformations associated with chloramphenicol use appears low; however, maternal treatment in late pregnancy may be associated with vascular collapse in the newborn infant. Therefore, chloramphenicol 15 mg/kg four times daily IV (maximum 1 g/dose) should be considered for treatment of tularemia in nonpregnant adults and children only when other recommended antimicrobial options have been exhausted. Serum drug concentrations should be carefully monitored during treatment.

#### Comparative Effectiveness

A systematic literature review of published individual tularemia cases worldwide compared outcomes for patients treated with fluoroquinolone, aminoglycoside, or tetracycline class monotherapy (*4*). Monotherapy was defined as treatment with a single effective antimicrobial and no other antimicrobials considered effective for tularemia. The fatality rate for patients who received tetracycline class monotherapy was low (2.3%), although slightly higher than that of the aminoglycoside or fluoroquinolone groups (1.1% and 1.2%, respectively). Among patients with primary pneumonic tularemia, the rates of thoracentesis or pleural effusion drainage and need for respiratory support were lower for those who received tetracycline class monotherapy compared with aminoglycoside or fluoroquinolone monotherapy; however, treatment bias and small case numbers in these groups limit interpretation. The rate of fever recurrence among patients who received tetracycline monotherapy was slightly higher (6.3%) than that of the aminoglycoside or fluoroquinolone groups (0% and 2.7%, respectively).

An analysis of U.S. surveillance data demonstrated that patients who received fluoroquinolones had increased adjusted odds of survival (adjusted odds ratio [aOR] = 5.3 [95% CI = 1.7–16.4]) compared with those who received an aminoglycoside, tetracycline, or no effective antimicrobial. Patients who received a tetracycline or aminoglycoside had similar elevated odds of survival (aOR = 4.9 [95% CI = 1.9–12.6] and aOR = 3.9 [95% CI = 1.05–14.7], respectively) compared with patients who received a different antimicrobial class or no effective antimicrobial. However, interpretation of these data is complicated by small numbers in individual treatment groups and wide CIs (*34*).

A review of tularemia case series publications described variable rates of treatment failure and relapse depending on the antibiotic used; however, time to treatment appeared to be a critical factor in determining outcome, regardless of the antibiotic used (*18*). When considering the use of tetracyclines versus fluoroquinolones for treatment of tularemia, the authors wrote, “In many patients, treatment failures and relapses are related to the site of infection (e.g., prosthetic infection), treatment delay higher than 2–3 weeks, and the occurrence of complications such as lymph node suppuration, rather than the antibiotic treatment administered.”

During an outbreak of *F. tularensis* subspecies *holarctica* (Type B) in Sweden, 29 hospitalized patients with severe pneumonic disease were evaluated, nine of whom also had bacteremia (*81*). The patients received either ciprofloxacin (n = four), levofloxacin (n = five), doxycycline (n = six), or a combination of these drugs with or without gentamicin (n = 14). One patient treated with doxycycline for 15 days relapsed 19 days after discharge but recovered after ciprofloxacin treatment. All five patients treated with levofloxacin monotherapy rapidly defervesced within 96 hours of treatment initiation and survived. The only death among this group occurred in a man aged 78 years with comorbidities and delayed treatment with ciprofloxacin and gentamicin (*81*).

In Central Anatolia, Turkey, a comparison of 139 cases of tularemia found no difference between the success rates of aminoglycoside and fluoroquinolone treatments (*82*). Another analysis of 142 cases of tularemia in northwestern Spain found that ciprofloxacin treatment resulted in fewer therapeutic failures (4.5% [one of 22]) when compared with streptomycin (23.4% [22 of 94]) and doxycycline (42.8% [six of 14]), although the small number of patients treated with doxycycline biased this analysis. This study also found fewer cases of sequelae among those treated with ciprofloxacin (*28*). An investigation of 177 tularemia cases in France found that patients treated with fluoroquinolones had a significantly higher rate of treatment failure (33% [14 of 43]) compared with those treated with doxycycline (9.9% [nine of 91]), although high failure rates might have been a result of treatment delays and heterogeneity in antimicrobial dosages and duration (*83*).

In a study assessing treatment of oropharyngeal tularemia in Turkey, no deaths were reported among 145 patients with Type B tularemia (*84*). The mean interval from onset of symptoms to diagnosis was 21 days, and 38% of patients overall experienced therapeutic failure, characterized by persistent or recurring fever, ongoing symptoms, or new or increased lymphadenopathy. Treatment duration was 10 days for aminoglycosides and 14 days for fluoroquinolones or tetracyclines. Among patients who experienced treatment failure, most (82%) had a delay of >14 days in initiating effective antimicrobials, and delays were associated with treatment failure regardless of antimicrobial class administered. Treatment failure rates were 29% (14 of 48) for fluoroquinolones, 32% (14 of 44) for aminoglycosides, and 51% (27 of 53) for doxycycline. Among the 48 patients treated with a fluoroquinolone, 37 received ciprofloxacin and 11 received moxifloxacin, with equivalent therapeutic success (*84*).

### Clinical Considerations for Treatment Recommendations

All forms of tularemia, including ulceroglandular, pneumonic, and typhoidal disease, can be treated with ciprofloxacin, levofloxacin, gentamicin, or doxycycline. Typically, it is not necessary to adjust treatment regimens based on a patient’s clinical manifestation, except for tularemia meningitis. General recommendations for treatment duration are presented (Table 1). Health care providers should use clinical judgment and consider extending treatment for patients with persistent fevers, abscesses, or other concerning signs.

For patients with severe infection, indicated by hemodynamic instability, end-organ dysfunction, need for respiratory support, or other signs of sepsis or severe illness, health care providers should consider treating initially with an aminoglycoside if possible because of the more robust clinical experience and published evidence of aminoglycoside use for severe disease. Health care providers also can consider prescribing combination therapy with two classes of effective antimicrobials (e.g., gentamicin plus ciprofloxacin or gentamicin plus doxycycline). Although evidence is minimal that initial treatment with two distinct classes of antimicrobials improves patient outcomes for severe tularemia (*4*,*85*), this approach may be considered in certain circumstances because of the potential need for empiric therapy against other infections, antimicrobial synergies, or differences in tissue penetrations.

When treatment delays occur, *F. tularensis* is more likely to establish itself in tissues, particularly lymph nodes. This increases the likelihood that additional surgical measures (e.g., lymphadenectomy or removal of infected joint prostheses) will be required. Bactericidal agents (e.g., ciprofloxacin) might be more effective than bacteriostatic agents in these situations. Although controlled studies in humans have not been conducted in recent decades, one comprehensive study of mice demonstrated that ciprofloxacin was more effective than doxycycline when treatment was delayed (*42*). Moreover, a study of cynomolgus macaques found that tissue bacterial clearance was more complete in animals treated with ciprofloxacin versus doxycycline (*38*). Multiple studies of Turkish patients with primarily oropharyngeal disease found that a delay of >2 weeks or ≥3 weeks in receiving appropriate antimicrobial treatment was associated with intranodal necrosis (*82*), therapeutic failure (*82*,*84*,*86*), and significantly increased recovery time (*84*). During an outbreak of ulceroglandular and typhoidal tularemia in Spain, in which the average time from symptom onset to diagnosis was 47.5 days, the success rate was highest for ciprofloxacin (95.5%) compared with streptomycin (76.6%) and doxycycline (57.1%) (*28*). Thus, for patients with a delay in treatment >2 weeks, health care providers should consider using a bactericidal agent (e.g., ciprofloxacin or gentamicin) over the bacteriostatic doxycycline or using 2-drug combination therapy (*18*,*82*,*86*).

Although rare, Jarisch-Herxheimer–like reactions have been reported after initiation of streptomycin treatment for tularemia (*80*,*87*). Patients with disseminated disease should be monitored after the first antimicrobial dose.

As always, general precautions for medication administration should be considered and shared with patients. For example, patients who take tetracyclines should drink a glass of water with each dose and remain upright for 30 minutes afterward to reduce the risk for esophageal irritation and ulceration (*88*). Calcium can inhibit absorption of certain tetracyclines and fluoroquinolones; thus, patients might need to avoid taking these medications with milk, yogurt, or calcium-fortified juice (*89*,*90*).

#### Neuroinvasive Tularemia

Central nervous system (CNS) infections including meningitis, focal lesions, and rhombencephalitis stemming from *F. tularensis* are rare but potentially fatal. One review identified 18 cases of neuroinvasive tularemia published in the English-language literature since 1950, including two described by the review authors (*14*). Before the advent of effective antimicrobials, nearly all patients with tularemia meningitis died (*91*). In contrast, 17 of the 18 patients recently summarized survived: 12 (66.7%) recovered without sequelae, four (22.2%) were discharged with sequelae, and one (5.6%) recovered but subsequently died 7 months later from lymphoma. The one fatal case identified in the review, a boy aged 1 year, did not receive effective antibiotics and died from diffuse hemorrhage and multisystem organ failure (*14*).

Health care providers have typically treated tularemia meningitis with at least two effective antimicrobial drugs, usually an aminoglycoside combined with chloramphenicol, a tetracycline, or a fluoroquinolone for ≥10 days (*14*,*91*–*95*). Although most patients have recovered with this type of regimen, a man aged 60 years treated with chloramphenicol and streptomycin experienced a relapse 6 days after discharge. He recovered completely after additional treatment with streptomycin and tetracycline (*94*). In two cases published separately, patients received only one effective antimicrobial: a boy aged 5 years who survived with no sequalae after receiving gentamicin for at least 16 days and a woman aged 28 years who recovered completely after treatment with ciprofloxacin (*14*,*96*). Although intrathecal aminoglycosides may sometimes be used for gram-negative meningitis, no records of this approach being applied for neuroinvasive tularemia were found.

Aminoglycosides are considered to have relatively poor cerebrospinal fluid (CSF) penetration, with an estimated AUC_CSF_/AUC_Serum_ of 0.2 among patients with uninflamed or mildly inflamed meninges (*97*). Nevertheless, patients with neuroinvasive tularemia have been treated successfully with gentamicin alone or in combination (*14*).

Fluoroquinolones have relatively robust CNS penetration levels (*98*,*99*). On the basis of an analysis of antimicrobials for anthrax meningitis, estimated geometric mean penetration ratios (AUC_CSF_/AUC_Plasma_) in patients with meningeal inflammation were 0.44 for ciprofloxacin and 0.38 for levofloxacin. AUC_CSF_/AUC_Plasma_ among patients with limited inflammation was 0.17 for doxycycline and 0.14 for amikacin (*100*).

Patients with neuroinvasive tularemia should be treated with combination gentamicin plus a fluoroquinolone (ciprofloxacin or levofloxacin). Gentamicin plus doxycycline may also be an acceptable alternative when fluoroquinolones are unavailable or contraindicated. Patients treated successfully for neuroinvasive tularemia have received an aminoglycoside for at least 10 days. Once gentamicin is discontinued and the patient is transitioned to an oral therapy, treatment should be continued for a total treatment duration of 21 days. As an alternative, chloramphenicol may be used in combination with gentamicin, a fluoroquinolone, or doxycycline; however, because of the potentially severe toxicity of chloramphenicol, this regimen should only be used when other antimicrobial options are limited. Health care providers should check drug-specific guidelines for chloramphenicol dosing for meningitis and serum drug concentration monitoring.

### Antimicrobial Pre- and Postexposure Prophylaxis of Tularemia for Adults and Children

In the event of an intentional release of *F. tularensis*, antimicrobial pre-exposure prophylaxis (PrEP) for first responders or health care providers caring for patients with tularemia is not considered necessary because of the low risk for person-to-person transmission of *F. tularensis*. Antimicrobial PrEP is also not recommended for members of the general public.

If an intentional release has occurred, persons likely exposed should be provided with PEP as soon as possible after the release, ideally within 48 hours of exposure. PEP monotherapy is recommended, with change to a new drug if engineered resistance is detected or patients develop clinical infection. Recommended first-line and alternative antimicrobial drugs for PEP after *F. tularensis* exposure are provided (Table 2).

First responders and health care providers who followed standard precautions (e.g., personal protective equipment [PPE]) while caring for patients with tularemia need not take PEP. Antimicrobial PEP or fever monitoring might be considered for first responders and health care providers who had breaches in PPE and substantial exposure while caring for patients with tularemia (e.g., direct contact with a patient’s ulcer without wearing gloves or performing intubation without wearing a surgical mask).

Response teams conducting environmental evaluations or other activities in an area of an intentional release of *F. tularensis* should use PPE recommended by the Environmental Protection Agency (EPA) (*101*) to reduce risk for infection from environmental contamination. If appropriate EPA-recommended PPE is not available and workers cannot avoid being in the area, antibiotic PrEP or PEP or fever monitoring after exposure may be considered.

Members of the general public are at risk for naturally acquired tularemia after tick or deer fly bites, lawn mowing, animal skinning, or other exposures. However, the overall risk after natural exposures is minimal because of the relatively low incidence of naturally acquired tularemia (0.064 per 100,000 population) (*102*). PEP is not typically recommended after a potential natural exposure, although PEP can be considered for hunters, pet owners, or other persons who had direct contact with an animal known to be infected.

Additional details on selection of specific antimicrobials for PEP, including the GRADE EtD framework underlying recommendations, are available (SupplementaryMaterial). Although evidence for fluoroquinolone use for PEP is particularly scant, fluoroquinolones are expected to be effective based on extrapolation from treatment data. In addition, evidence to inform optimal duration of PEP is limited.

Previously published tularemia guidelines recommended a 14-day duration for PEP with either doxycycline or ciprofloxacin (*7*,*103*). A study found that most rhesus macaques (*Macaca mulatta*) administered tetracycline prophylaxis for 13 days after aerosol exposure became ill once tetracycline was discontinued; however, *M. mulatta* are believed to be more susceptible to airborne *F. tularensis* than humans (*64*). In contrast, 2 g/day of tetracycline for 14 days was found to be effective for prophylaxis in humans; shorter durations were not tested (*64*). At the time of publication of these guidelines, no evidence has been found to suggest human prophylaxis with tetracyclines or fluoroquinolones of 7–13 days’ duration leads to greater risk for breakthrough infections compared with 14 days. Because fluoroquinolones are bactericidal and have been found to be more effective at eradicating *F. tularensis* from tissues than tetracyclines (*38*), a shorter PEP duration of 7 days for fluoroquinolones is recommended. The recommended PEP duration is 10–14 days for tetracycline class antibiotics and 10 days for azithromycin.

If supplies of first-line and alternative antimicrobials are unavailable, oral rifampin every 12 hours for 7 days may also be considered for PEP (5 mg/kg/dose for neonates, 10 mg/kg/dose for infants, children, and adolescents aged 1 month–17 years [maximum dose 600 mg], and 600 mg for adults). These doses are based on those used for prophylaxis of close contacts of patients with *Neisseria meningitidis* infection. Rifampin has low MICs in vitro (MIC50 0.5 *µ*g/mL and MIC90 1.0 *µ*g/mL) (*48*), is effective in mice infected via aerosol (*104*), and has been used occasionally to treat patients with tularemia (*105*–*107*). The utility of rifampin monotherapy is limited for most bacterial infections because of potential for development of resistance; however, the risk for developing resistance when used for prophylaxis in humans is likely very low because of minimal risk for transmission to other humans. Having an additional, distinct antimicrobial class available could be helpful after a bioterrorism attack, particularly if engineered resistance to fluoroquinolones or tetracyclines has been detected and inherent resistance to azithromycin is present.

#### Occupational Exposure

Laboratory workers who followed standard safety procedures (*1*,*7*,*108*,*109*) while handling specimens from patients with tularemia and who were not exposed by other means need not take PEP. Antimicrobial PEP should be considered for laboratory workers accidentally exposed to infectious materials through breaches in standard procedures. Monitoring for fever and other signs of infection is also an option instead of PEP for laboratory exposures, particularly if deemed to be low risk.

*F. tularensis* has been transmitted to humans from hares, rabbits, prairie dogs, voles, sheep, cats, dogs, and other animals (*3*,*10*,*110*). Thus, veterinary staff, animal control officers, herders, and other persons with frequent animal contact could be at risk for contracting tularemia from animals during a naturally occurring epizootic or after an intentional release of *F. tularensis*. The overall risk for transmission from ill animals to veterinary staff during routine care appears to be low; however, occupational infections after a needlestick injury and a necropsy have been reported (*111*,*112*). Depending on the perceived risk for exposure, PEP with a single drug or fever watch should be considered for persons after a needlestick injury, bite, scratch, or direct contact with secretions or body fluids from an animal with suspected or confirmed tularemia.

### Antimicrobial Treatment and Prophylaxis of Tularemia for Special Populations

#### Pregnancy and Tularemia

When selecting a tularemia treatment or prophylaxis regimen for pregnant patients, antimicrobial effectiveness should be prioritized, particularly in the setting of severe and complex disease. Clinicians also should consider available safety data when choosing an antimicrobial drug for use during pregnancy; however, fetal safety concerns should not prevent access to rapid treatment or prophylaxis for pregnant women during a tularemia outbreak. Appropriate treatment of infection is important to prevent adverse maternal, pregnancy, and infant outcomes.

Pregnancy results in multiple physiologic changes, including alterations in immunity, cardiovascular output, and renal function, as well as decreased lung capacity and gastrointestinal motility. These physiologic changes should be taken into consideration when selecting and administering antimicrobial tularemia treatment or prophylaxis for pregnant patients. Specifically, for certain antimicrobials, dose adjustments might be required in the later stages of pregnancy (late second through third trimesters), and the timing of transition from IV to oral administration might differ for pregnant patients. Furthermore, antimicrobial drugs have differences in transplacental passage, which might be considered in cases of suspected vertical transmission of tularemia.

Robust data on the management of pregnant women with tularemia and effectiveness of antimicrobial treatment to reduce adverse maternal, pregnancy, and infant outcomes are lacking. A recent systematic review identified 52 cases of tularemia during pregnancy worldwide. Although no maternal deaths were reported, a higher proportion of pregnancy losses occurred among mothers who did not receive any antimicrobial treatment for tularemia (16.7%) compared with those who did (10.5%) (*32*). Among those who received any antimicrobial treatment, the incidence of lymph node aspiration or excision was lower when at least one antimicrobial considered effective against *F. tularensis* was administered (47.8%) compared with those who received only antimicrobials not considered effective (80.0%). Two pregnancy losses occurred among mothers who received antimicrobial treatment: one who received penicillin monotherapy (*113*) and one who received a combination of penicillin and streptomycin. In the second case, treatment with streptomycin occurred >2 months after illness onset. A pathologic examination of the fetus found no evidence indicating that streptomycin therapy or tularemia contributed to fetal death (*114*). Because this systematic review was based solely on a collection of case reports, inherent biases were likely present. Regardless, these data suggest that pregnancy loss and other complications occur less frequently among pregnant women with tularemia who receive effective antimicrobial treatment than those who do not, similar to what is observed with other infectious diseases during pregnancy.

Without treatment, maternal–fetal transmission of *F. tularensis* might be possible, although the evidence is based on a single case report of a pregnant woman who did not receive effective treatment. The risk for possible maternal–fetal transmission with or without antimicrobial treatment is unknown. The one case of suspected congenital transmission reported in the literature involved a mother who developed ulceroglandular tularemia in the eighth month of pregnancy and experienced an intrauterine fetal demise 1 month after illness onset (*115*). Organisms consistent with *F. tularensis* were identified in placental and fetal tissues, suggesting that congenital transmission might have occurred.

Another case of possible congenital tularemia was recently identified in a neonate whose mother developed fever, sore throat, conjunctivitis, and cervical lymphadenopathy at 34 weeks’ gestation (B Nelson, University of Utah, unpublished data, 2025). During a cesarean delivery at 37 weeks, the mother’s fallopian tubes were removed and salpingitis was noted. Although the neonate was initially healthy, he was hospitalized for fever, CSF pleocytosis, and brain lesions at age 2 weeks, and a blood test was positive for *F. tularensis* via molecular testing. Subsequent testing of the fallopian tube tissue was positive for *F. tularensis* via immunohistochemistry and 16S polymerase chain reaction.

Although antimicrobial treatment has theoretical risks, tularemia is a potentially severe disease, and pregnant women should receive effective treatment or prophylaxis to reduce the risk for adverse maternal, pregnancy, and infant outcomes. When distinct options for antimicrobial classes are available, safety profiles of various antimicrobial drugs can guide selection of those that maximize benefit to the pregnant woman and her pregnancy and reduce risk for congenital transmission while minimizing potential fetal risk.

Multiple systematic reviews have examined the safety of fluoroquinolones and aminoglycosides during pregnancy. Three recent reviews found no evidence of an association between maternal fluoroquinolone use and adverse maternal or fetal outcomes (*116*–*118*). No clear association between maternal gentamicin exposure and birth defects has been documented clinically; however, adverse pregnancy outcomes (e.g., irreversible fetal ototoxicity) have been associated with streptomycin (*119*).

##### Treatment of Pregnant Women

For pregnant women with any form of tularemia, fluoroquinolones (i.e., ciprofloxacin or levofloxacin) or gentamicin are recommended for first-line treatment (Table 3) (SupplementaryMaterial). The dose and duration of fluoroquinolones should align with recommendations for the nonpregnant adult population; however, more frequent administration of ciprofloxacin or levofloxacin might be required because of increased renal clearance of fluoroquinolones during the latter half of pregnancy. Furthermore, because of the increased plasma volume and higher renal clearance among pregnant women, aminoglycoside drug concentrations should be checked as necessary to facilitate dose and duration adjustments. Streptomycin is not recommended for pregnant women because of its lack of availability and association with irreversible fetal ototoxicity.

Because of the limited experience with certain antimicrobial drugs during pregnancy and known complications with others (e.g., tetracycline), pregnant women should be prioritized for first-line agents. However, moxifloxacin, ofloxacin, amikacin, tobramycin, and doxycycline can be used as alternatives to treat pregnant patients if first-line options are unavailable.

Doxycycline is considered an alternative treatment option because of the more robust experience with other recommended antimicrobial drugs during pregnancy and possible risks of doxycycline use during pregnancy (e.g., pregnancy loss). Although certain published studies have not identified safety issues with doxycycline use during pregnancy (*120*), others have found increased risks for pregnancy loss and cardiovascular malformations (*121*–*123*). Moreover, because of the possibility of congenital transmission of *F. tularensis* and limited data on effectiveness of antimicrobial drugs for tularemia in pregnant women, bactericidal antimicrobials are preferred for treatment.

Dual therapy with distinct antimicrobial classes is recommended for treatment of severe tularemia during pregnancy. Specifically, gentamicin combined with either ciprofloxacin or levofloxacin is the preferred regimen. Because pregnant women have decreased gastrointestinal absorption and motility, and might not tolerate oral medications, parenteral administration is preferred over the oral route initially for moderate-to-severe tularemia; however, health care providers can consider discontinuing gentamicin and switching to oral administration of ciprofloxacin or levofloxacin when appropriate. As with other severe infections, if a preterm delivery (≤37 weeks) is anticipated for maternal or fetal indications within 7 days, corticosteroids (e.g., betamethasone and dexamethasone) are recommended for fetal benefit, in accordance with ACOG/SMFM guidelines (*124*).

Initial treatment with two distinct effective antimicrobial classes is recommended after a suspected bioterrorism attack because of the risk for engineered antimicrobial resistance; this is the same treatment as for nonpregnant adults. In addition to the first-line and alternative treatment options (Table 3), if supplies of these antimicrobial drugs are unavailable or patients have contraindications to these options, azithromycin 500 mg orally or IV for 10 days can be considered if the pathogen released is known to be *F. tularensis* subspecies *tularensis* (Type A) or *F. tularensis* subspecies *holarctica* (Type B) biovar I or biovar *japonica*.

##### PEP for Pregnant Women

For PEP, monotherapy is preferred for pregnant women (*122*), as for nonpregnant adults. The recommended antimicrobials for PEP for pregnant women are similar to those for nonpregnant adults, with the exception that doxycycline is an alternative rather than a first-line option (Table 4) (SupplementaryMaterial).

Azithromycin is also included as an alternative option for PEP because it is effective for most strains of *F. tularensis* and poses minimal risk to the fetus. If supplies of first-line and alternative antimicrobials have been depleted, oral rifampin (600 mg every 12 hours for 7 days) may also be considered as a third-tier option for PEP.

#### Neonates

Whether tularemia manifests or progresses differently in neonates compared with the general pediatric population is unclear. A systematic literature review did not identify any published reports of tularemia in neonates (*4*). However, a case of tularemia initially diagnosed as herpes simplex virus infection in an infant aged 6 weeks illustrates the potential challenges of distinguishing tularemia from more common infectious diseases diagnosed during the neonatal period (*125*). The infant had fever and a vesicular rash on the foot and was treated unsuccessfully with IV acyclovir. After laboratory testing identified *F. tularensis*, the infant was administered gentamicin and clarithromycin for 10 days; despite initial improvement, fever and vesicular rash recurred 2 weeks later. A second course of gentamicin for 14 days led to complete resolution of illness. Tularemia has been reported in older infants who had either oropharyngeal (*126*,*127*), ulceroglandular (*128*), glandular (*129*), or typhoidal (*130*) disease; all were successfully treated with gentamicin with or without additional antimicrobials, including doxycycline, ciprofloxacin, cephalosporins, macrolides, and rifampin.

An analysis of U.S. surveillance reports that included supplementary clinical data identified no neonates but included four infants with tularemia: a boy aged 2 months with typhoidal disease, a boy aged 9 months with pneumonic disease, a boy aged 10 months with ulceroglandular disease, and a girl aged 10 months with an unknown clinical manifestation (*34*) (K Kugeler, CDC, unpublished data, 2025). The boy aged 9 months became very ill and developed severe pediatric acute respiratory distress syndrome (*127*). After *F. tularensis* was cultured from bronchoalveolar lavage fluid on hospital day 21, gentamicin was initiated and he ultimately recovered. The girl aged 10 months had fever and gastrointestinal symptoms and did not receive an antibiotic effective for tularemia; she died 3 days after hospital admission. *F. tularensis* subspecies *holarctica* (Type B) was later cultured from lung tissue.

Neonates born to mothers with untreated or inadequately treated tularemia could potentially have congenital infection (*115*). Therefore, clinicians should weigh management options for these neonates, taking into consideration the extent and nature of the mother’s illness and the clinical status of the newborn. In multiple cases reported, neonates born to mothers with tularemia were reported to have had immunoglobulin G (IgG) antibodies against *F. tularensis* initially but became seronegative within 1 year, indicating they likely received passive transfer of maternal IgG and potentially had some protection against infection during the early weeks to months of life (*131*,*132*).

##### Treatment of Neonates

For these recommendations, neonates are defined as full-term infants aged ≤28 days or infants born premature who have reached 37–44 weeks postmenstrual age. For neonates of earlier postmenstrual age or with impaired renal function, health care providers should consult specialists if indicated and modify dosing as appropriate.

Gentamicin and ciprofloxacin are first-line agents as monotherapy for treatment of tularemia in neonates (Table 5). Levofloxacin, amikacin, tobramycin, and doxycycline can be used as alternative options. Chloramphenicol and streptomycin are third-tier options for treatment of tularemia in neonates and can be considered if other recommended antimicrobial options have been exhausted.

For ill-appearing neonates born to mothers with untreated or inadequately treated tularemia, clinicians should initiate presumptive treatment with gentamicin while pursuing additional diagnostic workup. These neonates should also be evaluated and treated for other, more common causes of neonatal sepsis as indicated.

##### PEP for Neonates

First-line agents for prophylaxis of neonates potentially exposed to *F. tularensis* are ciprofloxacin, levofloxacin, and doxycycline (Table 6). Ofloxacin is not typically used for neonates but can be considered if first-line antimicrobials are not available.

Healthy-appearing neonates born to mothers who received a full course of treatment for tularemia can be monitored initially but need not be prescribed antimicrobial prophylaxis. For healthy-appearing neonates born to mothers with untreated or inadequately treated tularemia, clinicians can consider watchful waiting versus antimicrobial prophylaxis. Factors to consider include severity of the mother’s illness, systemic versus localized maternal infection, and whether and to what extent the mother has received antimicrobial treatment.

##### Additional Considerations for Neonates

Multiple important factors should be considered when selecting antimicrobials for treatment and prophylaxis of tularemia among neonates. Many antimicrobials, including tobramycin and ofloxacin, have not been specifically evaluated or approved for use in neonates; therefore, clinicians must balance the risks and benefits of use.

Certain neonates regularly experience gastroesophageal reflux (spitting up), making it difficult to administer a full medication dose orally. Moreover, neonates regularly ingest breast milk or formula, which have substantial amounts of calcium and other minerals that can inhibit absorption of oral fluoroquinolones and tetracycline (*89*,*90*). Thus, antimicrobials for treatment of ill neonates with tularemia should be administered intravenously when possible to ensure that the full desired dose is successfully administered. If IV access cannot be obtained, doxycycline can be administered orally to neonates for treatment of tularemia if a nasogastric tube has been placed. This approach also can be used to transition neonates from IV to oral antimicrobials for treatment of tularemia once they have clinically improved. Ciprofloxacin oral suspension should not be administered via nasogastric or gastric tube because of its tendency to clog the tubing (*133*); crushed tablets mixed with liquid can be administered instead. Antimicrobial prophylaxis can be administered orally to neonates when possible.

#### Lactating Mothers and Breastfeeding Infants

Although no studies have directly assessed the presence of *F. tularensis* in breast milk of mothers infected with tularemia, there are no known reports of *F. tularensis* transmission from mother to child through breast milk. A 1947 publication described an Austrian woman who “breastfed her 4-month-old child while ill with tularemia without the infant falling ill” (translated from German) (*134*).

The risk for *F. tularensis* transmission through ingestion of breast milk is likely little to none. In general, mothers with tularemia and mothers taking antimicrobial prophylaxis after exposure to *F. tularensis* can continue to breastfeed their infants if able. One notable exception is if a lactating mother has an ulcer on the breast, because direct transmission could occur if an infant’s skin or oral mucosa come into contact with the ulcer. A lactating mother with a breast ulcer should be advised to cover the ulcer with gauze and surgical tape for the duration of treatment and until the ulcer has healed or developed a scab. If the ulcer is adjacent to or overlying the nipple, mothers should avoid breastfeeding on that side for the duration of treatment but continue regular expression of breast milk via hand or mechanical pump and discard the milk.

When selecting antimicrobials for lactating mothers who require tularemia treatment or prophylaxis, generally the recommendations align with those for nonlactating mothers. However, clinicians should consider transmission of the drug or metabolites to the infant via breast milk when prescribing an antibiotic. The CDC guidelines for treatment and prophylaxis of plague include a detailed description of penetration of aminoglycosides, tetracyclines, and fluoroquinolones in breast milk (*135*). These antimicrobial classes typically produce low concentrations in breast milk and have an acceptable safety profile. Short-term use of tetracycline is acceptable for lactating mothers; however, prolonged or repeat courses should be avoided because of a theoretical risk for dental enamel staining or bone deposition (*136*). Furthermore, lactating mothers should be advised that minocycline can cause black discoloration of breast milk (*137*).

Breast milk concentrations of azithromycin are typically low; however, infants should be monitored for vomiting, diarrhea, and candidiasis because azithromycin can affect the gastrointestinal flora. Evidence has indicated that maternal use of macrolide antibiotics during the first 2 weeks of breastfeeding might increase the risk for infantile hypertrophic pyloric stenosis, although this has not been confirmed (*138*). Breastfeeding infants whose mothers are being treated with chloramphenicol might experience vomiting and somnolence and have a theoretical risk for aplastic anemia (*139*).

#### Geriatric Patients

Geriatric patients, defined as persons aged ≥65 years, are more likely to develop pneumonic and typhoidal tularemia (*4*,*34*,*140*) and experience a higher fatality rate compared with younger patients, regardless of clinical manifestation (*4*,*34*). A systematic review of cases published worldwide found a higher fatality rate for geriatric patients who received tetracycline monotherapy (7.0% versus 0.6% of patients aged <60 years), fluoroquinolone monotherapy (6.0% versus 0%), or a combination of effective antimicrobial classes (2.0% versus 0.9%). In contrast, no deaths were reported among geriatric patients who received aminoglycoside monotherapy (*4*) (CA Nelson, D Meaney-Delman, S Fleck-Derderian, PS Mead, CDC; J Winberg, Alaka`ina Foundation; unpublished data, 2025).

An analysis of U.S. surveillance data found no deaths among patients of any age who were treated with aminoglycoside monotherapy, although small case numbers in this category limit interpretation. Geriatric patients had slightly higher CFRs when treated with tetracycline monotherapy (1.6% versus 0.4% of patients aged <60 years), fluoroquinolone monotherapy (1.9% versus 0.7%), or a combination of effective antimicrobial classes (2.9% versus 0%) (*32*).

The recommended first-line and alternative antimicrobials for treatment and prophylaxis of tularemia do not differ for geriatric patients compared with adult patients aged <65 years (Table 1). However, when selecting antimicrobials, geriatric patients might have greater risk for certain adverse events associated with specific agents. The risk for tendinitis, tendon rupture, QT interval prolongation, aortic aneurysm, aortic dissection, and other adverse events associated with fluoroquinolones is higher among elderly patients (*141*–*143*). Of note, risk variability within the fluoroquinolone class is substantial; for example, moxifloxacin is associated with twice the risk for arrhythmia and a higher risk for dysglycemia compared with ciprofloxacin and levofloxacin (*144*). The CNS effects of fluoroquinolones, including agitation, insomnia, anxiety, dizziness, confusion, and weakness also are potentiated in older adults and could lead to substantial difficulties, particularly during a mass prophylaxis scenario when hundreds or thousands of potentially exposed older adults might require prophylaxis (*141*). The risk for dizziness is higher for levofloxacin than ciprofloxacin (*145*,*146*).

The likelihood of aminoglycoside-related nephrotoxicity and neurotoxicity has been demonstrated to be higher among patients of advanced age (*42*,*142*). Moreover, for tetracycline class antibiotics, vestibular toxicity appears to be more likely among geriatric patients taking minocycline. However, whether doxycycline and tetracycline are also associated with increased risk for vestibular symptoms among this population is unknown (*143*).

Careful consideration must also be given to underlying conditions and medication interactions because geriatric patients are more likely to have comorbidities and polypharmacy. Notably, fluoroquinolones can enhance the anticoagulant effect of warfarin and hypoglycemic effects of agents that lower blood glucose levels (*144*). Aminoglycosides have greater risk for nephrotoxicity in patients taking long-term nonsteroidal anti-inflammatory drugs (NSAIDS) or undergoing immunosuppressive therapy (*61*). Tetracycline class antibiotics can enhance the effects of vitamin K antagonists (e.g., warfarin), and absorption might be decreased in persons taking antacids, calcium, antiepileptic drugs (e.g., carbamazepine), and other medications (*147*). For current, comprehensive information on medication use and polypharmacy in older adults, health care providers should refer to the American Geriatrics Society Beers Criteria, which are updated regularly (*148*).

#### Patients With Immunocompromise

Human immunity to tularemia develops primarily through cellular components, but humoral immunity also contributes (*1*,*149*). Notably, substantial heterogeneity exists among patients with immunocompromise, which clinicians should consider when managing tularemia. For example, the Advisory Committee on Immunization Practices further categorizes patients with altered immunocompetence into primary (intrinsic) and secondary (acquired) immunodeficiency with varying severity and effects (*150*).

Patients with immunocompromise diagnosed with tularemia have been successfully treated with the same antimicrobials used to treat immunocompetent patients. An analysis of tularemia cases from U.S. surveillance data collected during 2006–2021 identified 131 of 1,163 patients (11.3%) with immunocompromise (*34*). Pneumonic and typhoidal manifestations occurred more frequently among patients with immunocompromise compared with those without known immunocompromise (CA Nelson, D Meaney-Delman, S Fleck-Derderian, PS Mead, CDC; J Winberg, Alaka`ina Foundation; unpublished data, 2025). Among 100 patients with immunocompromise with a known outcome, three died, although none of these patients received an effective antimicrobial for tularemia. Analysis indicated that immunocompromised patients had similar odds of survival compared with immunocompetent patients (OR = 0.9 [95% CI = 0.3–2.7]) in this data set (*34*).

In a review of literature published during 1996–2019, a total of 17 cases of tularemia were identified in persons with immunocompromise, two of whom were children (*151*). The leading cause of immunosuppression was immunosuppressive therapy (n = 14 [82%]), including seven solid organ transplant recipients, three hematopoietic stem cell transplant recipients, and four patients with an autoimmune disease (*151*). In addition, two patients were living with AIDS, and one had chronic granulomatous disease. Of the 17 patients, eight (48%) had pneumonic tularemia, five (29%) had typhoidal, and four (24%) had glandular or ulceroglandular disease (*150*). Ten patients with immunocompromise included in the review received monotherapy and recovered: three were treated with ciprofloxacin, three with doxycycline, two with levofloxacin, and one with an unknown fluoroquinolone; three patients experienced relapse after initial treatment (*151*). The single fatal case described in the review was a man aged 43 years who developed a high fever and lethargy on day 2 after a bone marrow transplant; gentamicin was added to his antimicrobial regimen on day 9, but he experienced cardiac arrest and died on day 14 (*151*).

Treatment recommendations for patients with immunocompromise do not differ from those for the general population (Table 1). As with any patient, health care providers should recognize the potential for polypharmacy with resultant drug–drug interactions and adjust antimicrobials accordingly. Patients with tularemia who are immunocompromised should be carefully monitored, treatment duration should be potentially extended, and drainage procedures (e.g., lymphadenectomy) considered if indicated based on clinical judgment.

#### Patients with Obesity or Who Are Underweight

Volume of distribution, metabolism, and elimination of antimicrobials can be affected by body weight. Adjustments in dose might be necessary and are most important for drugs with a narrow therapeutic index (e.g., aminoglycosides). These adjustments are independent of those related to alterations in hepatic or renal function.

Ciprofloxacin should be dosed at the upper end of the dosing range for patients with obesity (body mass index [BMI] ≥30). No dosing adjustment is necessary for levofloxacin or moxifloxacin. Similarly, tetracyclines do not require dose adjustment for patients with obesity or who are underweight (*152*).

Aminoglycosides have a narrow therapeutic index and require therapeutic drug monitoring to avoid toxicity. For patients with a BMI ≥30, initial dosing should be based on adjusted body weight (ABW), calculated as ideal body weight (IBW) plus 40% of excess body weight: ABW = IBW + ([total body weight − IBW] x 0.4) (*152*). Of note, once daily dosing of aminoglycosides might not be appropriate for patients with severe obesity (BMI ≥40). For patients who are underweight (BMI <18.5), initial dosing should be based on total body weight.

For children, less specific dosing information is available. Evidence indicates that aminoglycosides can be dosed according to ABW (as for adults) for children with obesity, whereas all other agents should be dosed according to total body weight (*119*–*121*). In all cases, total dose for children should not exceed the maximum dose for an adult (*122*).

### Response to *F. tularensis* Release as a Biologic Weapon or Large-Scale Natural Outbreaks

Intentional release of *F. tularensis* during a bioterrorism attack could result in a mass-exposure event leading to considerable public health consequences and disruption of U.S. society. If an intentional release is suspected because of a disclosed threat, unexpected illnesses among the public, or other factors, health care providers and local public health practitioners should communicate immediately with state and Federal health and security authorities to coordinate response and determine threat credibility.

Efficient response to an intentional release of *F. tularensis* would require rapid identification of the source of transmission, ability to distinguish cases of tularemia from other diseases including those caused by other potential bioweapons (e.g., *Yersinia pestis* or *Bacillus anthracis*), testing the released strain for engineered antimicrobial resistance, and rapid deployment of appropriate PEP and treatment. A bioterrorism attack might involve aerosol release of *F. tularensis*, contamination of water or food, or other mechanisms of spread.

Initial signs and symptoms of *F. tularensis* infection could range from mild and nonspecific manifestations to an acute onset of influenza-like illness or sepsis. Recognition of an unusual increase in suspected tularemia cases or influenza-like illness, whether locally or nationally, would suggest a possible intentional release or large-scale natural outbreak and *F. tularensis*–specific diagnostic testing should be conducted as soon as possible. If a bioterrorism attack is suspected, antimicrobial susceptibility testing also should be performed.

*F. tularensis* strains released during a bioterrorism attack might be engineered for antimicrobial resistance. Until antimicrobial susceptibility patterns are known, health care providers should treat symptomatic patients with suspected exposure with two distinct classes of antimicrobial drugs, at least one of which is considered first-line, to increase the likelihood that at least one effective drug is administered.

Antimicrobial PEP should be considered only for asymptomatic persons likely exposed during a bioterrorism attack. PrEP is not recommended. Assessment of eligibility for receipt of prophylaxis would be determined by Federal, state, and local public health agencies based on the context of the bioterrorism attack, risk for individual exposure, available resources, and an ethical framework similar to other bioterrorism countermeasures (*153*,*154*).

In the wake of a large-scale emergency response to an intentional release or naturally occurring outbreak, a substantial number of patients might receive delayed or inadequate treatment, increasing the risk for complications such as prolonged fevers, pulmonary nodules, or lymph node suppuration. Moreover, in rare cases, *F. tularensis* has been found to persist in tissues. *F. tularensis* was cultured from the axillary lymph node of a boy who underwent excisional biopsy 49 days after onset of fever and lymphadenopathy; before the procedure, the patient had received amoxicillin clavulanate but no antimicrobial drug effective for tularemia (*155*). In addition, one case of longstanding pulmonary *F. tularensis* infection spanning multiple years has been described recently in the literature (*156*). Although prolonged infection appears to be rare, it could develop in certain patients who are exposed to *F. tularensis* and receive insufficient antimicrobial treatment.

### Personal Protective Equipment

Person-to-person transmission of tularemia is rare, and the only known infection transmitted from a living person was associated with direct inoculation through the skin (*9*). The systematic literature review and other evidence informing development of these guidelines (see Methods) revealed no known cases of transmission between humans via the respiratory route. Moreover, a case series published in 2020 described nine health care providers who did not develop tularemia after substantial exposures to infected patients, underscoring the low risk for transmission from patients to health care providers. Certain health care providers included in the case series had performed what often are considered aerosol-generating procedures (e.g., bronchoscopy, intubation, and bone sawing during replacement of an infected joint); these health care providers wore face masks during the procedures, and certain providers wore goggles, although none wore N95-style filtering facepiece respirators (*157*). The very low risk for occupational infection is further supported by lack of reported infections among health care providers who cared for hundreds of patients with tularemia in Northern European hospitals, despite formal reporting systems for such incidents (*158*) (AF Johansson, MD PhD, Umeå University, Sweden, personal communication, 2024).

Standard precautions should be used when caring for all patients with tularemia. Additional protection with masks, goggles, face shields, and combinations of each should be selected according to the need anticipated for the task performed. Health care providers should use PPE to protect the eyes, nose, and mouth when performing procedures (e.g., lymph node aspiration) that could generate sprays or splashes to these areas (*7*,*159*). Moreover, during aerosol-generating procedures, health care providers should wear one of the following: a face shield that fully covers the front and sides of the face, a mask with attached shield, or a mask and goggles (in addition to gloves and gown) (*160*).

In accordance with CDC recommendations, health care providers should use transmission-based precautions when providing care to patients with undiagnosed respiratory infections (*161*,*162*). Precautions should be tailored to the likely etiologic agents and the patient’s clinical syndrome. Once the specific infection has been identified, health care providers can adjust precautions accordingly. Because there is no known evidence of human-to-human *F. tularensis* transmission via respiratory aerosols, particulate-filtering facepiece respirators (e.g., N95 respirators) are not necessary when providing routine care for patients with pneumonic tularemia.

Although human-to-human *F. tularensis* transmission via respiratory aerosols has not been documented, exposure to aerosolized *F. tularensis* in the environment and laboratory is a well-described risk factor for pneumonic tularemia. First responders and emergency response teams conducting environmental response activities might be at risk for infection because of environmental contamination. These workers should wear appropriate PPE in accordance with Federal, state, and local public health guidance and the EPA Quick Response Guide for *F. tularensis* (*163*).

Because *F. tularensis* is highly infectious, laboratory workers handling cultures are at risk for occupational exposure and infection (*108*,*164*–*169*). If tularemia is suspected in a patient, health care providers should notify laboratory workers who will be processing clinical samples from that patient. Laboratory safety measures for handling *F. tularensis* should be followed (*1*,*7*,*109*,*111*).

## Future Directions

Tularemia is a complicated infectious disease with many remaining knowledge gaps. Additional research related to prevention, diagnosis, antimicrobial treatment, and adjunct treatments can help advance the field and prepare for future outbreaks. As new data become available regarding treatment and prophylaxis of *F. tularensis*, these recommendations might need to be updated.

A safe and effective vaccine could be useful for persons who are at risk for tularemia because of their avocation or occupation, including first responders and other emergency response personnel. The first tularemia vaccines developed were live-attenuated formulations using the LVS strain. Although LVS vaccines have reduced certain forms of laboratory-acquired infections, they are not currently licensed in the United States because of limited effectiveness for all forms of tularemia and incomplete protection against large inocula of *F. tularensis* (*170*–*172*). In recent decades, research into the development of an effective tularemia vaccine has continued, including use of additional live attenuated formulations, inactivated strains, subunit vaccines, and DNA vaccines (*173*–*175*).

Research on additional testing modalities to rapidly diagnose tularemia is also critical because tularemia can have protean clinical manifestations, and delays in recognition and treatment are clearly associated with worse outcomes. Moreover, tests for rapid identification of *F. tularensis* antimicrobial sensitivities and detection of engineered resistance would be helpful to improve emergency response and optimize resource allocation after an intentional release. Development of these tests is ongoing; however, additional comprehensive research is needed.

Although multiple antimicrobial classes are effective for treatment of tularemia, many questions remain about the relative efficacy of the various classes and individual antimicrobials within each class. Furthermore, additional investigations are imperative to address the needs for all populations at risk for tularemia, including pregnant and lactating women, infants, children, patients with immunocompromise, and geriatric patients.

## Limitations

The recommendations in this report are subject to at least four limitations. First, the evidence supporting these recommendations is based in part on systematic reviews of tularemia case reports and case series. These sources are considered low-quality evidence with a high risk for bias in reported data. Randomized controlled trials of various antimicrobials for treatment and prophylaxis of tularemia have not been conducted in recent decades and are exceptionally challenging because of the sporadic nature of tularemia outbreaks. Second, comparisons of survival and complication rates by antimicrobial treatment were limited by small numbers of patients in various treatment groups and the frequency of combination therapy administered to patients. Third, animal data helped inform these recommendations but do not fully represent human tularemia infections. The GRADE EtD framework was used to improve transparency and elucidate the rationale for decision points within these guidelines. Finally, the use of *F. tularensis* as a bioweapon is based on intelligence reports; because there has been no known use in actual conflict, treatment and policy recommendations are speculative and based on clinical experience with naturally occurring infection.

## Conclusion

*F. tularensis* causes naturally occurring infection, with a history of large outbreaks in warzones, and poses a bioterrorism risk because of its low infectious dose. Although tularemia can be treated with antimicrobials, the disease can be fatal or lead to substantial complications. Early recognition and administration of effective antimicrobial drugs are important for all patients and will be critical during a large-scale outbreak of tularemia. Moreover, multiple antimicrobials are effective for PEP and can save lives if distributed rapidly to patients exposed to *F. tularensis*.

Fluoroquinolones, aminoglycosides, and tetracyclines are the mainstays of antimicrobial therapy for tularemia, specifically ciprofloxacin, levofloxacin, gentamicin, or doxycycline. Dual treatment with a combination of effective antimicrobial classes is recommended for patients with severe disease. In the wake of a bioterrorism attack, initial treatment with two distinct effective antimicrobial classes is recommended because of the risk for engineered antimicrobial resistance. Treatment can be narrowed once patients have improved and additional information about the specific *F. tularensis* strain released becomes available.

These guidelines use newer evidence generated in the past 2 decades to provide recommendations for management of tularemia resulting from naturally occurring or bioterrorism-related transmission. These recommendations can aid health care providers during clinical care of patients with tularemia and can be used to develop and strengthen emergency response plans and preparedness at the local, state, and Federal levels.

## Footnotes

* Standards of Ethical Conduct for Employees of the Executive Branch, 5 CFR Sect. 2635.

† Extended-interval dosing involves administration of a higher weight-based dose at an extended interval (i.e., every 24 hours for most patients or longer intervals for patients with renal dysfunction).
